# Efficient tandem electroreduction of nitrate into ammonia through coupling Cu single atoms with adjacent Co_3_O_4_

**DOI:** 10.1038/s41467-024-48035-4

**Published:** 2024-04-29

**Authors:** Yan Liu, Jie Wei, Zhengwu Yang, Lirong Zheng, Jiankang Zhao, Zhimin Song, Yuhan Zhou, Jiajie Cheng, Junyang Meng, Zhigang Geng, Jie Zeng

**Affiliations:** 1 Hefei National Research Center for Physical Sciences at the Microscale, University of Science and Technology of China, Hefei, 230026 Anhui PR China; 2 Institute of High Energy Physics, Chinese Academy of Sciences, 100049 Beijing, PR China; 3 Department of Physics, University of Science and Technology of China, Hefei, 230026 Anhui PR China; 4 CAS Key Laboratory of Strongly-Coupled Quantum Matter Physics, University of Science and Technology of China, Hefei, 230026 Anhui PR China; 5 Key Laboratory of Surface and Interface Chemistry and Energy Catalysis of Anhui Higher Education Institutes, Department of Chemical Physics, University of Science and Technology of China, Hefei, 230026 Anhui PR China; 6 School of Chemistry & Chemical Engineering, Anhui University of Technology, Ma’anshan, 243002 Anhui PR China

**Keywords:** Electrocatalysis, Pollution remediation, Materials for energy and catalysis

## Abstract

The nitrate (NO_3_^−^) electroreduction into ammonia (NH_3_) represents a promising approach for sustainable NH_3_ synthesis. However, the variation of adsorption configurations renders great difficulties in the simultaneous optimization of binding energy for the intermediates. Though the extensively reported Cu-based electrocatalysts benefit NO_3_^−^ adsorption, one of the key issues lies in the accumulation of nitrite (NO_2_^−^) due to its weak adsorption, resulting in the rapid deactivation of catalysts and sluggish kinetics of subsequent hydrogenation steps. Here we report a tandem electrocatalyst by combining Cu single atoms catalysts with adjacent Co_3_O_4_ nanosheets to boost the electroreduction of NO_3_^−^ to NH_3_. The obtained tandem catalyst exhibits a yield rate for NH_3_ of 114.0 mg_NH3_ h^−1^ cm^−2^, which exceeds the previous values for the reported Cu-based catalysts. Mechanism investigations unveil that the combination of Co_3_O_4_ regulates the adsorption configuration of NO_2_^−^ and strengthens the binding with NO_2_^−^, thus accelerating the electroreduction of NO_3_^−^ to NH_3_.

## Abstract (web summary)

An optimal catalyst for nitrate electroreduction should satisfy the simultaneously optimized adsorption of intermediates. Here, the authors report a tandem electrocatalyst by combining Cu single atoms with Co_3_O_4_ nanosheets, enhancing the binding with NO_2_^−^, thus promoting nitrate electroreduction to NH_3_.

## Introduction

As one of the nitrogen-containing species, nitrate (NO_3_^−^) widely exists in industrial and agricultural wastewater with a high concentration, mainly caused by the emission of low-level nuclear waste and intensive usage of fertilizers^1–3^. Excessive NO_3_^−^ has significantly threatened ecological balance, inducing acid rain and photochemical smog^4^. Additionally, NO_3_^−^ in human body is easily converted into toxic nitrite (NO_2_^−^), leading to serious health issues^5^. Among the methods for removing NO_3_^−^, electroreduction process using renewable electricity is regarded as an appealing technology under mild conditions^6–9^. The controllable products including nontoxic nitrogen (N_2_) and valuable ammonia (NH_3_) could be obtained after NO_3_^−^ electroreduction^10–12^. Since NH_3_ is a fundamental chemical compound and a promising green hydrogen carrier, NO_3_^−^ electroreduction into NH_3_ instead of N_2_ is more desirable. Taken together, it is highly imperative to achieve efficient electroreduction of NO_3_^−^ into NH_3_ from the perspective of environmental protection and sustainable NH_3_ synthesis.

In view of the multiple nitrogen-containing intermediates (e.g. *NO_3_, *NO_2_, and *NO) involved in the NO_3_^−^ electroreduction, an optimal catalyst should satisfy the simultaneously optimized adsorption of intermediates. The moderate binding energy of intermediates serves as one of the key factors for efficient NO_3_^−^ electroreduction into NH_3_^13,14^. Classically, given that the coordination of N atom in NO_3_^−^ is saturated by three O atoms, *NO_3_ tends to bond with active sites through O atoms. Whereas, *NO_2_ is preferentially adsorbed on active sites through N and O atoms. As for *NO, N atom in *NO is inclined to connect with active sites. The variation of adsorption configurations renders great difficulties in the simultaneous optimization of binding energy for the intermediates. A typical instance is Cu-based electrocatalysts which have been reported extensively for NO_3_^−^ electroreduction^14–20^. Though Cu-based electrocatalysts benefit NO_3_^−^ adsorption, one of the key issues lies in the accumulation of NO_2_^−^, resulting in the rapid deactivation of catalysts and sluggish kinetics of the subsequent hydrogenation steps for NH_3_ production^15,17^. However, it remains a grand challenge to design an efficient catalyst to satisfy the simultaneously optimized adsorption of intermediates with different configurations.

Herein, we report a tandem electrocatalyst by combining Cu single atoms anchored on N-doped carbon with adjacent Co_3_O_4_ nanosheets (denoted as Co_3_O_4_/Cu_1_-N-C) to boost the electroreduction of NO_3_^−^ to NH_3_. The obtained Co_3_O_4_/Cu_1_-N-C catalyst exhibits a remarkable yield rate for NH_3_ of 114.0 mg_NH3_ h^−1^ cm^−2^, which exceeds the previous values for all of the reported Cu-based catalysts. Mechanism investigations unveil that the combination of Co_3_O_4_ regulates the adsorption configuration of NO_2_^−^ and strengthens the binding with NO_2_^−^, thus accelerating the electroreduction of NO_3_^−^ to NH_3_.

## Results

### Catalyst synthesis and characterizations

**Fig. 1:**
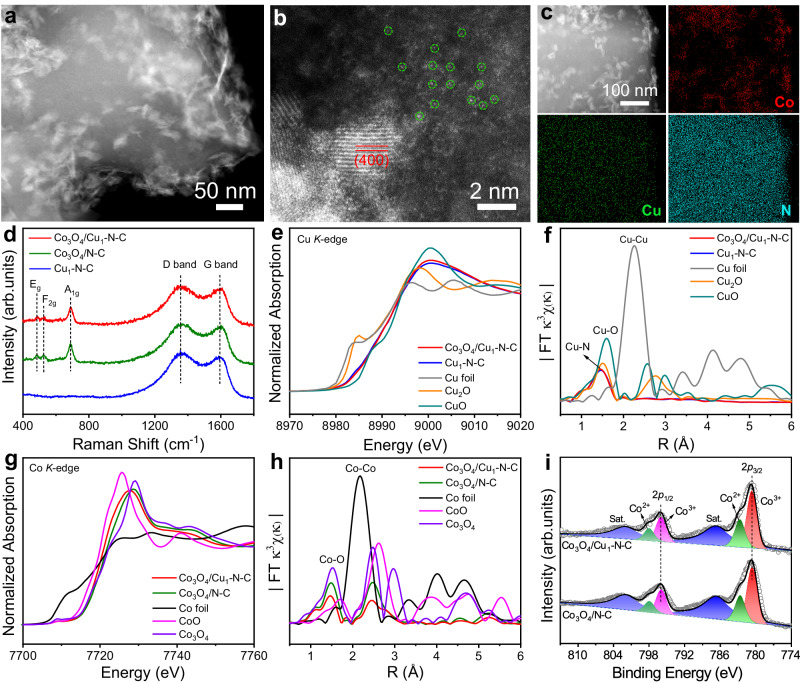
Structural characterizations. **a** HAADF-STEM image, **b** aberration-corrected HAADF-STEM image, and **c** EDS elemental mappings of Co_3_O_4_/Cu_1_-N-C. **d** Raman spectra of Co_3_O_4_/Cu_1_-N-C, Cu_1_-N-C, and Co_3_O_4_/N-C. **e** Cu *K*-edge XANES spectra and **f** EXAFS spectra for Co_3_O_4_/Cu_1_-N-C, Cu_1_-N-C, Cu foil, Cu_2_O, and CuO. **g** Co *K*-edge XANES spectra and **h** EXAFS spectra for Co_3_O_4_/Cu_1_-N-C, Co_3_O_4_/N-C, Co foil, CoO, and Co_3_O_4_. **i** Co 2*p* XPS spectra for Co_3_O_4_/Cu_1_-N-C and Co_3_O_4_/N-C.

Co_3_O_4_/Cu_1_-N-C catalyst was synthesized by adding sodium borohydride to the mixture containing Cu single-atom catalysts and cobalt nitrate. Cu single atoms dispersed on N-doped carbon (denoted as Cu_1_-N-C) were prepared via pyrolyzing Cu-doped ZIF-8 at 900 °C under Ar atmosphere (Supplementary Figs. 1 and 2). Figure 1a shows the high-angle annular dark field scanning transmission electron microscopy (HAADF-STEM) image of Co_3_O_4_/Cu_1_-N-C. As displayed in the high resolution TEM (HRTEM) image and the corresponding selected area electron diffraction pattern (SAED), Co_3_O_4_ nanosheets were successfully deposited on the surface of Cu_1_-N-C (Supplementary Fig. 3). Figure 1b shows the aberration-corrected HAADF-STEM image of Co_3_O_4_/Cu_1_-N-C. The lattice fringes with an interplanar spacing of 0.201 nm were ascribed to the (400) facet of Co_3_O_4_. Besides, abundant Cu single atoms were observed around Co_3_O_4_ nanosheets. Based on energy-dispersive X-ray spectroscopy (EDS) elemental mapping, Co, Cu, and N elements were uniformly distributed throughout the whole structure (Fig. 1c). The uniform distribution of Cu sites and Co_3_O_4_ species constituted the adjacent catalytic centers. The metal content of Cu and Co in Co_3_O_4_/Cu_1_-N-C were determined to be 0.60 wt% and 4.70 wt%, respectively, by inductively coupled plasma-optical emission spectroscopy analysis (ICP-OES). For comparison, Co_3_O_4_ nanosheets dispersed on N-doped carbon (denoted as Co_3_O_4_/N-C) were prepared with a similar synthetic procedure of Co_3_O_4_/Cu_1_-N-C except for the addition of Cu precursor (Supplementary Fig. 4). Figure 1d shows the Raman spectra for Co_3_O_4_/Cu_1_-N-C, Cu_1_-N-C, and Co_3_O_4_/N-C. All of the Raman spectra displayed two peaks located at 1356 and 1591 cm^−1^, assigned to the D band and G band of graphite carbon, respectively^21^. The similar intensity ratios of D band to G band (I_D_/I_G_) for the three samples indicated that the carbon support possessed similar degree of structural disorder (Supplementary Fig. 5). Compared with Cu_1_-N-C, three distinguishable peaks located at 482, 527, and 689 cm^−1^ were observed for both Co_3_O_4_/Cu_1_-N-C and Co_3_O_4_/N-C, corresponding to E_g_, F_2g_, and A_1g_ vibration modes of Co_3_O_4_ crystals, respectively^22^. The structure of graphite carbon supports was further confirmed by X-ray diffraction patterns (Supplementary Fig. 6). Figure 1e shows the Cu *K*-edge X-ray absorption near edge structure (XANES) spectra of Co_3_O_4_/Cu_1_-N-C and Cu_1_-N-C. Obviously, the energy absorption edge profiles for both Co_3_O_4_/Cu_1_-N-C and Cu_1_-N-C were located between those of CuO and Cu_2_O, elucidating that the valence state of Cu species in the two catalysts were between +1 to +2. As shown in Fig. 1f, a dominant peak at 1.93 Å was observed in the extended X-ray absorption fine structure (EXAFS) spectra of Cu *K*-edge for Co_3_O_4_/Cu_1_-N-C and Cu_1_-N-C, which were attributed to the Cu-N bond. The absence of Cu-Cu bond in the two catalysts further confirmed the atomic dispersion of Cu species. Besides, the EXAFS fitting results indicate that the coordination numbers of Cu-N shell in both Co_3_O_4_/Cu_1_-N-C and Cu_1_-N-C were approximately 4.0 (Supplementary Fig. 7 and Table 1). After the deposition of Co_3_O_4_ nanosheets, the coordination structure of Cu single atoms (CuN_4_) in Co_3_O_4_/Cu_1_-N-C was unchanged. Besides, the wavelet transformed EXAFS (WT-EXAFS) spectra of Co_3_O_4_/Cu_1_-N-C and Cu_1_-N-C also confirmed the Cu-N bonding in the two catalysts (Supplementary Fig. 8). For the Co *K*-edge XANES spectra, the edge energy for both Co_3_O_4_/Cu_1_-N-C and Co_3_O_4_/N-C were similar to that for Co_3_O_4_ reference (Fig. 1g). Figure 1h shows that the Co-O coordination in Co_3_O_4_/Cu_1_-N-C and Co_3_O_4_/N-C were approximate to that in Co_3_O_4_, certifying the similar coordination structure of Co_3_O_4_ species in the two catalysts (Supplementary Fig. 9 and Table 2). Figure 1i shows the Co 2*p* X-ray photoelectron spectroscopy (XPS) spectra. Specifically, the peaks at 798.0, 782.7, 796.1, and 780.8 eV in Co_3_O_4_/Cu_1_-N-C and Co_3_O_4_/N-C corresponded to Co^2+^ 2*p*_1/2_, Co^2+^ 2*p*_3/2_, Co^3+^ 2*p*_1/2_, and Co^3+^ 2*p*_3/2_, respectively^23^. The indiscernible shift of Co 2*p* peaks demonstrated that Cu_1_-N-C as the support did not significantly affect the valence state of Co.

### Catalytic performance toward NO_3_^−^ electroreduction

**Fig. 2:**
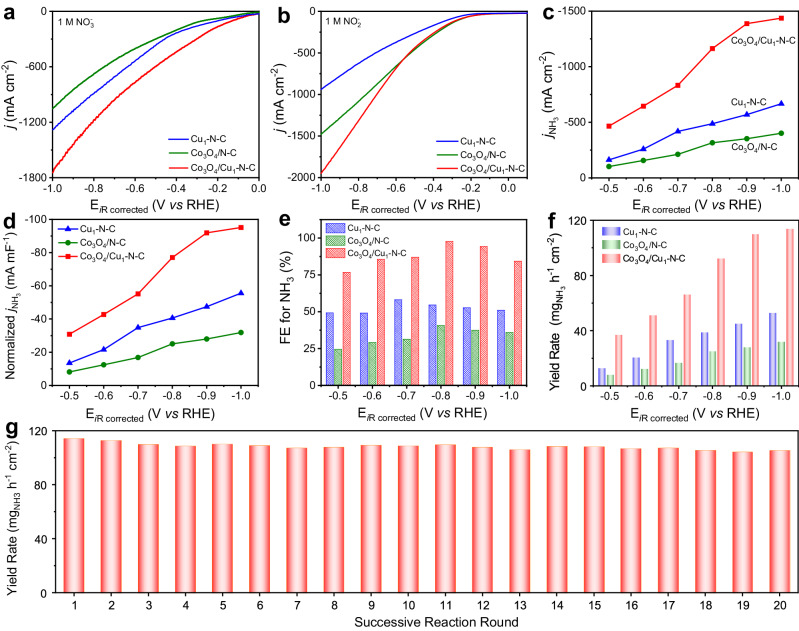
Catalytic performance. LSV curves of Cu_1_-N-C, Co_3_O_4_/N-C, and Co_3_O_4_/Cu_1_-N-C with (**a**) 1 M NO_3_^−^ and (**b**) 1 M NO_2_^−^. **c** *j*_NH__3_, **d** normalized *j*_NH__3_ based on *C*_dl_, **e** FE for NH_3_, and **f** yield rate for NH_3_ of Cu_1_-N-C, Co_3_O_4_/N-C, and Co_3_O_4_/Cu_1_-N-C at different applied potentials with 1 M NO_3_^−^. **g** Yield rate for NH_3_ of Co_3_O_4_/Cu_1_-N-C at −1.0 V *vs* RHE under 20 rounds of successive reactions. The solution resistance was determined to be 4.4 ± 0.2 ohm in the electrolytes by potentiostatic electrochemical impedance spectroscopy.

The catalytic performance of the catalysts was investigated in a three-electrode H-type cell toward NO_3_^−^ electroreduction (Supplementary Fig. 10). The concentration of NH_3_ product was quantified by the indophenol blue method (Supplementary Fig. 11). To preliminarily explore the process of tandem catalysis, we conducted the linear sweep voltammetry (LSV) curves of Co_3_O_4_/Cu_1_-N-C, Cu_1_-N-C, and Co_3_O_4_/N-C with 1 M NO_3_^−^/NO_2_^−^, respectively. As shown in Fig. 2a, the current density of Cu_1_-N-C in the presence of NO_3_^−^ was higher than that of Co_3_O_4_/N-C, suggesting that Cu_1_-N-C possessed higher activity toward NO_3_^−^ electroreduction. Whereas, Co_3_O_4_/N-C exhibited a larger current density relative to Cu_1_-N-C in NO_2_^−^ (Fig. 2b). The superior activity of Co_3_O_4_ species toward NO_2_^−^ electroreduction was further demonstrated by the higher Faradaic efficiency (FE) and yield rate for NH_3_ of Co_3_O_4_/N-C in NO_2_^−^ electroreduction relative to Cu_1_-N-C (Supplementary Fig. 12). Considering that NO_2_^−^ is one of the vital intermediates, the combination of Cu_1_-N-C and Co_3_O_4_ would couple the separate functions of Cu sites and Co_3_O_4_ species for the sequential reduction of NO_3_^−^ and NO_2_^−^. As expected, Co_3_O_4_/Cu_1_-N-C displayed the highest current density among the three catalysts in the electrolyte of NO_3_^−^. Besides, the tremendous discrepancy of the LSV curves of Co_3_O_4_/Cu_1_-N-C in 1 M KOH with/without NO_3_^−^ also implied the superior activity of Co_3_O_4_/Cu_1_-N-C toward NO_3_^−^ electroreduction (Supplementary Fig. 13).

Figure 2c provides the partial current density for NH_3_ (*j*_NH__3_) of Co_3_O_4_/Cu_1_-N-C, Cu_1_-N-C, and Co_3_O_4_/N-C at various applied potentials toward NO_3_^−^ electroreduction. The *j*_NH__3_ of Co_3_O_4_/Cu_1_-N-C exceeded those of Cu_1_-N-C and Co_3_O_4_/N-C. Especially, at −1.0 V *vs* reversible hydrogen electrode (RHE), the *j*_NH__3_ of Co_3_O_4_/Cu_1_-N-C reached −1437.5 mA cm^−2^, which was 2.2 times and 3.6 times as high as that of Cu_1_-N-C and Co_3_O_4_/N-C, respectively. Moreover, the normalized *j*_NH__3_ based on double-layer capacitance (*C*_dl_) for Co_3_O_4_/Cu_1_-N-C was the largest among the three catalysts, indicating the highest intrinsic activity for Co_3_O_4_/Cu_1_-N-C toward NO_3_^−^ electroreduction (Fig. 2d and Supplementary Fig. 14). In addition, the FE for NH_3_ of Co_3_O_4_/Cu_1_-N-C was higher with respect to the other two counterparts at all applied potentials (Fig. 2e). Especially, Co_3_O_4_/Cu_1_-N-C achieved the maximum FE for NH_3_ of 97.7% at −0.8 V *vs* RHE. Furthermore, at −1.0 V *vs* RHE, the yield rate of NH_3_ for Co_3_O_4_/Cu_1_-N-C reached up to 114.0 mg_NH3_ h^−1^ cm^−2^, which exceeded all of the reported value for Cu-based catalysts^14,16,18,19,24–30^ (Fig. 2f and Supplementary Table 3). The yield rate of NH_4_^+^ in the electrolyte after the electroreduction process was also determined by ^1^H nuclear magnetic resonance (NMR) analysis, which was approximated to the results detected via the indophenol blue method (Supplementary Fig. 15 and Table 4). Other liquid and gaseous products including NO_2_^−^, NH_2_OH, NO, NO_2_, N_2_O, H_2_, and N_2_ for Co_3_O_4_/Cu_1_-N-C were also measured (Supplementary Figs. 16–18). NH_3_ were the only main product after NO_3_^−^/NO_2_^−^ electroreduction (Supplementary Table 5). Besides, the FE for NH_3_ of Co_3_O_4_/Cu_1_-N-C with NO_3_^−^ concentrations ranging from 10 mM to 500 mM all exceeded 91.2%, indicating a wide tolerance range for the concentration of NO_3_^−^ (Supplementary Fig. 19). The durability of Co_3_O_4_/Cu_1_-N-C was examined by 20 rounds of successive reactions. The negligible decay of the yield rate demonstrated the satisfactory durability of Co_3_O_4_/Cu_1_-N-C (Fig. 2g). The Raman and XAFS measurements for Co_3_O_4_/Cu_1_-N-C after the electrolysis indicated that the Co_3_O_4_ species and Cu-N bonding were preserved (Supplementary Figs. 20 and 21). The stability of Cu single atoms in Co_3_O_4_/Cu_1_-N-C during the electrolysis was further explored by in situ EXAFS measurements, indicating that Cu atoms remained the atomically dispersed state in Co_3_O_4_/Cu_1_-N-C during the NO_3_^−^ electroreduction (Supplementary Figs. 22 and 23).

To further clarify the synergy effect of Co_3_O_4_ on the conversion of NO_2_^−^, we conducted a series of control experiments. The catalytic performance of other metal oxides (such as FeO_x_, CuO_x_, and NiO_x_) dispersed on N-doped carbon toward NO_2_^−^ electroreduction were all lower than that over Co_3_O_4_/N-C, suggesting the inferior ability of these metal oxide to facilitate NO_2_^−^ reduction (Supplementary Figs. 24 and 25). In addition, the loading amount of Co_3_O_4_ on Cu_1_-N-C was vital to the efficient conversion of the accumulated NO_2_^−^ (Supplementary Fig. 26). Besides, the simply physical mixing of Cu_1_-N-C and Co_3_O_4_/N-C could not sufficiently assure the spatial couple of the adjacent sites, thereby limiting the effective hydrogenation of NO_2_^−^ into NH_3_ during NO_3_^−^ electroreduction (Supplementary Fig. 27). We also exclude the possible ammonia contamination from the self-electrolysis of Co_3_O_4_/Cu_1_-N-C, electrolyte, and carbon paper, respectively (Supplementary Fig. 28). Besides, the catalytic activity of N-doped carbon was much lower compared with that of Co_3_O_4_/Cu_1_-N-C (Supplementary Fig. 29). The possible interference of Co single atoms on Cu_1_-N-C support could be considered insignificant to the catalytic performance of Co_3_O_4_/Cu_1_-N-C (Supplementary Figs. 30–33). The electroreduction of NO_3_^−^ was also affected by the diffusion of reactants (Supplementary Fig. 34). Moreover, ^15^NO_3_^−^ isotopic labeling measurements for Co_3_O_4_/Cu_1_-N-C was conducted with ^1^H NMR analysis. Only typical doublet peaks attributed to ^15^NH_4_^+^ were collected with ^15^NO_3_^−^ as the N source whereas the triplet peaks of ^14^NH_4_^+^ were detected with ^14^NO_3_^−^ as the N source (Supplementary Fig. 35). These results indicated that the NH_3_ detected in the electrolyte originated from the electroreduction of NO_3_^−^.

### Mechanistic study on NO_3_^−^ electroreduction

**Fig. 3:**
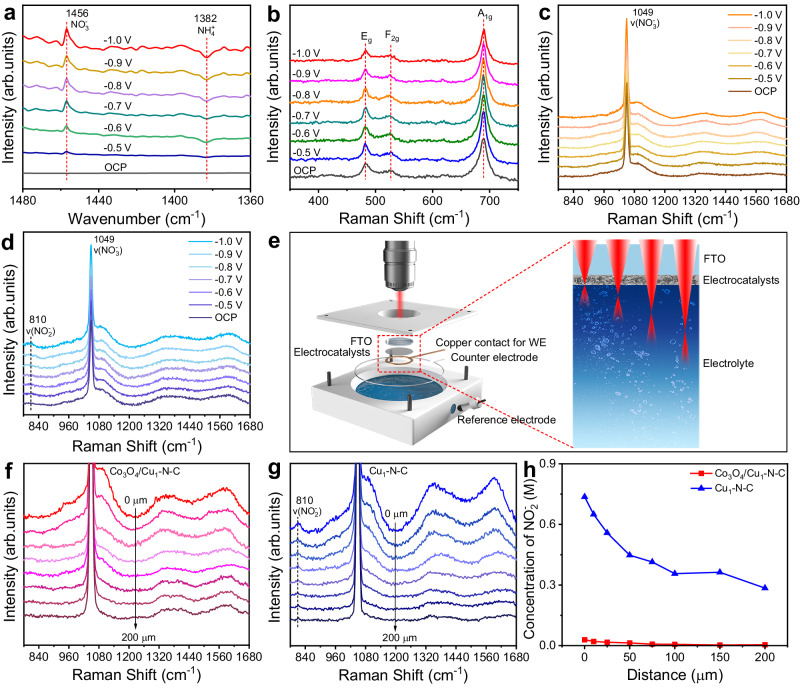
In situ characterizations. **a** In situ FTIR spectra for Co_3_O_4_/Cu_1_-N-C from OCP to −1.0 V *vs* RHE in 1 M NO_3_^−^. **b** In situ Raman spectra for Co_3_O_4_/Cu_1_-N-C from OCP to −1.0 V *vs* RHE in 1 M NO_3_^−^. In situ Raman spectra for (**c**) Co_3_O_4_/Cu_1_-N-C and (**d**) Cu_1_-N-C from OCP to −1.0 V *vs* RHE in 1 M NO_3_^−^. **e** Scheme of the designed Raman cell for detecting from the surface of catalysts to the electrolyte bulk. In situ Raman spectra for (**f**) Co_3_O_4_/Cu_1_-N-C and (**g**) Cu_1_-N-C at −0.8 V *vs* RHE in 1 M NO_3_^−^ with different distances ranging from 0 to 200 μm. **h** The calculated concentration of NO_2_^−^ for Co_3_O_4_/Cu_1_-N-C and Cu_1_-N-C with different distances ranging from 0 to 200 μm.

To gain more insight into the catalytic process of NO_3_^−^ electroreduction over Co_3_O_4_/Cu_1_-N-C, we conducted in situ electrochemical Fourier transform infrared spectroscopy (FTIR) and Raman spectroscopy to monitor the reaction process (Supplementary Figs. 36 and 37). Figure 3a displays the in situ FTIR spectra of Co_3_O_4_/Cu_1_-N-C at applied potentials from OCP to −1.0 V *vs* RHE. The negative peaks at 1382 cm^−1^ were ascribed to the consumption of NO_3_^−^ species^3^. In addition, the emergence of positive peaks located at 1456 cm^−1^ confirmed that NH_4_^+^ was generated during the NO_3_^−^ electroreduction^31^. Besides, two peaks at 1541 and 1508 cm^−1^ were detected, which were assigned to the vibration band of *NO and *NOH, respectively (Supplementary Fig. 38). Figure 3b shows the in situ Raman spectra of Co_3_O_4_/Cu_1_-N-C at all applied potentials. The peaks corresponding to E_g_, F_2g_, and A_1g_ vibration modes of Co_3_O_4_ remained unchanged, suggesting that the Co_3_O_4_ species was stable during NO_3_^−^ electroreduction. During the NO_3_^−^ electroreduction, only the peak at 1049 cm^−1^ was observed for Co_3_O_4_/Cu_1_-N-C at all applied potentials, assigned to the symmetric stretching vibration of NO_3_^−^ (Fig. 3c). In the case of Cu_1_-N-C, apart from the signal of NO_3_^−^, a new peak at 810 cm^−1^ ascribed to the bending vibration of NO_2_^−^ gradually appeared as the applied potential increased, indicating the accumulation of NO_2_^−^ for Cu_1_-N-C during NO_3_^−^ electroreduction (Fig. 3d). To further probe the variation of local concentration for NO_2_^−^ near the surface of the catalysts, we designed a Raman cell that allows Raman laser to detect from the surface of catalysts to the electrolyte bulk. The Raman laser was designed to be incident from the back of catalysts to diminish the interference from the strong absorbance of NO_3_^−^ in the electrolyte. As illustrated in Fig. 3e, the electrocatalysts were deposited on fluorine tin oxide-coated glass (FTO) as the working electrode (WE). The distance from the laser beam to electrode surface is controlled by the mechanical sample stage. Figure 3f, g display the in situ Raman spectra of Co_3_O_4_/Cu_1_-N-C and Cu_1_-N-C at −0.8 V *vs* RHE when the laser beam was positioned 0 to 200 μm away from the surface of catalysts, respectively. With the increment of the distance between the focal plane of laser and the surface of catalysts, the signal intensity of graphite carbon for the catalysts gradually decreased. A noticeable peak at 810 cm^−1^ assigned to NO_2_^−^ arose near the surface of Cu_1_-N-C. Whereas, the signal of NO_2_^−^ for Co_3_O_4_/Cu_1_-N-C was negligible, which was independent of the distance. Furthermore, we determined the local concentration of NO_2_^−^ near the surface of catalysts based on the integrated areas of NO_2_^−^ and NO_3_^−^, taking the ratio of integrated areas for 1 M NO_2_^−^ and 1 M NO_3_^−^ solutions as a correction factor (Supplementary Fig. 39). As the laser beam was set further far away from the surface of catalysts into the electrolyte, the concentration of NO_2_^−^ for Cu_1_-N-C gradually decreased from 0.74 to 0.29 M (Fig. 3h). This trend indicates that the NO_2_^−^ generated at Cu_1_-N-C/electrolyte interface diffused into the electrolyte due to the sluggish reduction of NO_2_^−^. Clearly, the concentration of NO_2_^−^ for Co_3_O_4_/Cu_1_-N-C was much lower relative to Cu_1_-N-C, manifesting the facilitated reduction of NO_2_^−^ with the favor of Co_3_O_4_.

**Fig. 4:**
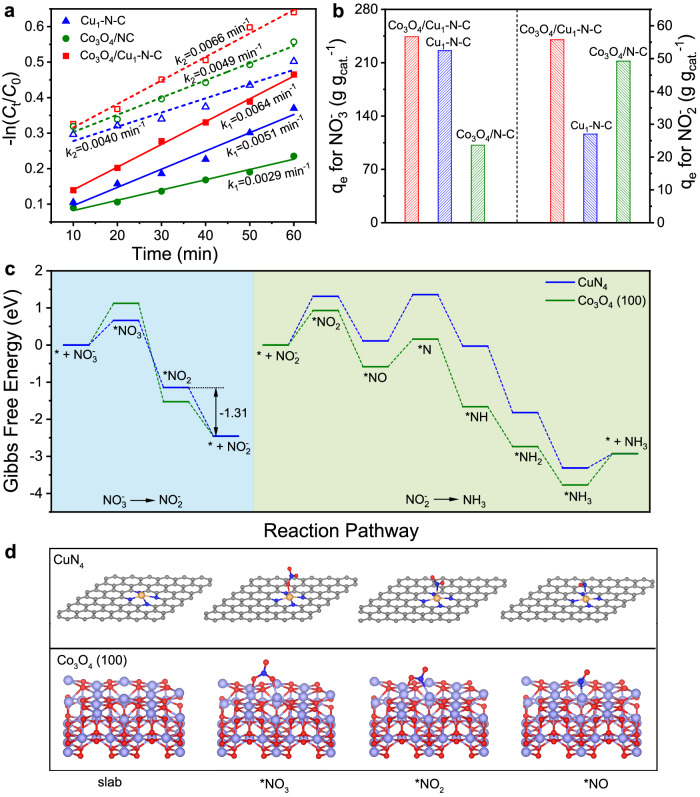
Mechanistic study on NO_3_^−^ electroreduction. **a** Linearized pseudo first-order kinetic profiles of Co_3_O_4_/Cu_1_-N-C, Cu_1_-N-C, and Co_3_O_4_/N-C in 1 M NO_3_^−^/NO_2_^−^, respectively. **b** Adsorption capacities of Co_3_O_4_/Cu_1_-N-C, Cu_1_-N-C, and Co_3_O_4_/N-C for NO_3_^−^/NO_2_^−^. **c** Free energy diagram of NO_3_^−^ electroreduction over CuN_4_ and Co_3_O_4_ (100) slabs. * represents an adsorption site. **d** Structure models of key intermediates on CuN_4_ and Co_3_O_4_ (100) slabs. The gray, blue, red, yellow, and purple spheres represent C, N, O, Cu, and Co atoms, respectively.

To further understand the synergetic role of Cu_1_-N-C and Co_3_O_4_ in the catalytic process, we calculated the rate constants for NO_3_^−^ electroreduction (*k*_1_) and NO_2_^−^ electroreduction (*k*_2_), respectively (Fig. 4a). The concentration of residual NO_3_^−^ after the electroreduction process was quantified by UV-Vis spectrophotometry (Supplementary Fig. 40). Compared with Co_3_O_4_/N-C, the larger *k*_1_ value of Cu_1_-N-C suggests that Cu_1_-N-C was more favorable for the conversion of NO_3_^−^ to NO_2_^−^, but the smaller *k*_2_ value shows the slower kinetics for NO_2_^−^ reduction. Accordingly, excessive NO_2_^−^ would be desorbed into the electrolyte for Cu_1_-N-C. Notably, the highest *k*_1_ and *k*_2_ of Co_3_O_4_/Cu_1_-N-C manifested the simultaneous acceleration of the conversion of NO_3_^−^ to NO_2_^−^ and NO_2_^−^ to NH_3_. Besides, the tafel slopes of Co_3_O_4_/Cu_1_-N-C, Co_3_O_4_/N-C, and Cu_1_-N-C in 1 M NO_3_^−^ and 1 M NO_2_^−^ imply that the combination of Co_3_O_4_ with Cu_1_-N-C facilitate the kinetics of NO_3_^−^ and NO_2_^−^ reduction during the catalytic process (Supplementary Fig. 41). Figure 4b shows the adsorption capacities (q_e_) of Co_3_O_4_/Cu_1_-N-C, Cu_1_-N-C, and Co_3_O_4_/N-C for NO_3_^−^ and NO_2_^−^, respectively. It is obvious that Co_3_O_4_/Cu_1_-N-C exhibited the largest q_e_ for both NO_3_^−^ and NO_2_^−^ among the three catalysts. As a consequence, combining Cu_1_-N-C with Co_3_O_4_ was conducive to the conversion of both NO_3_^−^ and NO_2_^−^.

The density functional theory (DFT) calculations were conducted to further interpret the reaction mechanism of NO_3_^−^ electroreduction over Co_3_O_4_/Cu_1_-N-C catalysts. Based on the results of structural analysis, we adopted CuN_4_ and Co_3_O_4_ (100) slabs as the models to calculate the Gibbs free energies (*G*) for each step involved in NO_3_^−^ electroreduction, respectively (Supplementary Fig. 42). After the structure optimization, *NO_3_ would be adsorbed on CuN_4_ with O atom. As presented in Fig. 4c, the Gibbs free-energy changes (Δ*G*) for NO_3_^−^ adsorption over CuN_4_ is much lower than that over Co_3_O_4_ (100), indicating the stronger binding of NO_3_^−^ over CuN_4_. Nevertheless, the Δ*G* of *NO_2_ desorption over CuN_4_ is −1.31 eV, which could be more thermodynamically favorable than the reduction of *NO_2_ to *NO (−1.20 eV). In this regard, the desorption of *NO_2_ would give rise to the accumulation of NO_2_^−^ over CuN_4_, which was consistent with the high yield for NO_2_^−^ over Cu_1_-N-C in NO_3_^−^ electroreduction (Supplementary Fig. 17). As for the conversion of NO_2_^−^, *NO_2_ would be adsorbed on CuN_4_ through N atom whereas *NO_2_ could be connected with Co_3_O_4_ (100) through N and O atoms after the structure optimization, leading to the lower Δ*G* for NO_2_^−^ adsorption over Co_3_O_4_ (100) (Fig. 4d). In addition, the relative high Δ*G* for *H adsorption over CuN_4_ and Co_3_O_4_ revealed the weak adsorption of *H, indicating that the occurrence of competitive H_2_ evolution could be depressed (Supplementary Figs. 43 and 44). As a result, Co_3_O_4_ would regulate the adsorption configuration of NO_2_^−^ and possesse an easier binding with NO_2_^−^, facilitating the reduction of NO_2_^−^ to NH_3_.

## Discussion

In summary, we developed a highly efficient catalyst by coupling the separate functions of Cu_1_-N-C and Co_3_O_4_ for the sequential reduction of NO_3_^−^ to NO_2_^−^ and NO_2_^−^ to NH_3_. The obtained Co_3_O_4_/Cu_1_-N-C catalyst exhibited a superior yield rate for NH_3_ of 114.0 mg_NH3_ h^−1^ cm^−2^, which exceeded all of the reported values for Cu-based catalysts. The mechanism investigations unveiled that the combination of Co_3_O_4_ regulated the adsorption configuration of NO_2_^−^ and strengthened the binding with NO_2_^−^, thus accelerating the electroreduction of NO_3_^−^ to NH_3_. This work offers a novel guideline for the construction of highly efficient tandem catalysts toward NO_3_^−^ electroreduction.

## Methods

### Chemicals and materials

Zinc nitrate hexahydrate (Zn(NO_3_)_2_·6H_2_O, 99.0%), 2-methyl imidazole (2-MeIM, 99.0%), copper(II) acetate monohydrate (Cu(COOCH_3_)_2_·H_2_O, 99.0%), cobalt nitrate hexahydrate (Co(NO_3_)_2_·6H_2_O, 99.0%), iron nitrate nonahydrate (Fe(NO_3_)_3_·9H_2_O, 98.5%), nickel nitrate hexahydrate (Ni(NO_3_)_2_·6H_2_O, 98.0%), copper nitrate trihydrate (Cu(NO_3_)_2_·3H_2_O, 99.0%), methanol (99.5%),ethanol (99.5%), potassium nitrate (KNO_3_, 99.0%), potassium nitrite (KNO_2_, 97.0%), potassium hydroxide (KOH, 85%), ammonium sulfate ((NH_4_)_2_SO_4_, 99.0%), sodium hydroxide (NaOH, ≥96.0%), salicylic acid (C_7_H_6_O_3_), sodium hypochlorite solution (NaClO, available chlorine 5.2% of aqueous solution), trisodium citrate dihydrate (C_6_H_5_Na_3_O_7_·2H_2_O), sodium nitroferricyanide dihydrate (C_5_FeN_6_Na_2_O·2H_2_O), hydrochloric acid (HCl, 12 mol L^−1^), sulfamic acid (99.5%), p-aminobenzenesulfonamide (98.0%), N-(1-Naphthyl) ethylenediamine dihydrochloride (98.0%), phosphoric acid (H_3_PO_4_, ≥85.0%), and 1-propanesulfonic acid 3-(trimethylsilyl) sodium salt (DSS) were purchased from Sinopharm Chemical Reagent Co. Ltd. Glyoxylic acid solution (C_2_H_2_O_3_, 50 wt%), dimethyl sulfoxide-*d*_6_ (DMSO-*d*_6_, 99.9atom% D), (^15^NH_4_)_2_SO_4_ (99.0 atom% ^15^N), and K^15^NO_3_ (99.0 atom% ^15^N) were purchased from Aladdin Chemistry Co., Ltd (Shanghai, China). Bipolar membrane (TRJBM) were purchased from Beijing Tingrun Membrane Technology Development Co., Ltd (Beijing, China). The deionized (DI) water was produced using a Millipore Milli-Q grade, with a resistivity of 18.2 MΩ cm. All of the chemicals were used without any further purification.

### Instrumentations

TEM images were taken using a Hitachi HT7700 transmission electron microscope at an acceleration voltage of 100 kV. HAADF-STEM and the corresponding EDS elemental mapping were carried out on a Talos F200X field-emission transmission electron microscope operated at an accelerating voltage of 200 kV using Mo-based TEM grids. Aberration-corrected HAADF-STEM images were carried out on Themis Z field-emission transmission electron microscope operating at an accelerating voltage of 300 kV using Mo-based TEM grids. XRD patterns were collected using a Rikagu MiniFlex X-ray diffractometer with Cu-Kα radiation (λ = 1.54059 Å). ICP-OES (Avio 220 MAX, PerkinElmer) analysis was employed to measure the concentration of metal species. XPS measurements were performed using a Kratos Axis supra+ diffractometer with Al-Kα radiation. The Raman spectra were conducted via LabRAM HR Evolution (Horiba) Raman system with a 532 nm excitation laser. The absorbance data was measured on a UV-vis spectrophotometer (Agilent Technologies, Cary 60). The in situ FTIR spectra were acquired by a Nicolet iS50 FTIR spectrometer with a built-in MCT detector.

### Synthesis of Cu_1_-N-C

A mixture of Zn(NO_3_)_2_·6H_2_O (5.6 mmol) and Cu(COOCH_3_)_2_·H_2_O (0.28 mmol) was dissolved in 80 mL of methanol, which was subsequently added into 80 mL of methanol containing 3.70 g of 2-MeIM. Then the mixed solution was kept at 25 °C for 12 h. The as-obtained precipitate (denoted as Cu-doped ZIF-8) was separated by centrifugation and washed subsequently with methanol for five times, and finally dried at 65 °C under vacuum overnight. Next, the obtained Cu-containing derivative of ZIF-8 was heated to 900 °C with a heating rate of 5 °C min^−1^ in a tube furnace and kept at 900 °C under flowing Ar gas for 3 h. After the tube furnace was naturally cooled to room temperature, Cu_1_-N-C was obtained and directly used as the catalyst without further treatment. For comparison, Co single atoms anchored on N-doped carbon (denoted as Co_1_-N-C) and Co single atoms anchored on Cu_1_-N-C (denoted as Co_1_Cu_1_-N-C) were obtained via pyrolyzing the Co-doped ZIF-8 and Cu/Co-doped ZIF-8, respectively. Co-doped ZIF-8 and Cu/Co-doped ZIF-8 prepared with the similar procedure with that of Cu_1_-N-C except the Co(NO_3_)_2_·6H_2_O and the mixture of Co(NO_3_)_2_·6H_2_O and Cu(COOCH_3_)_2_·H_2_O as the metal precursors, respectively.

### Synthesis of Co_3_O_4_/Cu_1_-N-C and Co_3_O_4_/N-C

160 mg of Cu_1_-N-C was dispersed in 20 mL of ethanol by sonication for 30 min. Afterwards, 5 mL of H_2_O containing 45 mg of Co(NO_3_)_2_·6H_2_O was added into the above solution, which was maintained in an ice-water bath for 1 h with vigorous stirring. Then, 40 mL of freshly prepared NaBH_4_ (100 mg) with ice-cold H_2_O was added dropwise into the above suspension, followed by further stirring for 1 h. The as-obtained precipitate was separated by filtration and washed subsequently with water for five times. Finally, Co_3_O_4_/Cu_1_-N-C was obtained by being dried at 65 °C under vacuum overnight. Co_3_O_4_/N-C was prepared as a comparison with the similar procedure with that of Co_3_O_4_/Cu_1_-N-C except for the addition of N-doped carbon instead of Cu_1_-N-C. N-doped carbon was prepared with a similar synthetic procedure with that of Cu_1_-N-C without the addition of Cu(COOCH_3_)_2_·H_2_O. For comparison, other metal oxides including FeO_x_, CuO_x_, and NiO_x_ dispersed on N-doped carbon (denoted as FeO_x_/N-C, CuO_x_/N-C, and NiO_x_/N-C, respectively) were prepared with the similar procedure with that of Co_3_O_4_/N-C except for the addition of Fe(NO_3_)_3_·9H_2_O, Cu(NO_3_)_2_·3H_2_O, and Ni(NO_3_)_2_·6H_2_O as the metal precursors, respectively (denoted as FeO_x_/N-C, CuO_x_/N-C, and NiO_x_/N-C, respectively).

### X-ray absorption fine structure (XAFS) measurements

The XAFS spectra at Cu *K-*edge and Co *K*-edge were performed at 1W1B beamline of Beijing Synchrotron Radiation Facility and BL11B beamline of Shanghai Synchrotron Radiation Facility. The data were obtained in ambient conditions under fluorescence mode for Cu *K-*edge and transmission mode for Co *K*-edge, respectively.

The ATHENA module and ARTEMIS codes in the IFEFFIT software packages were employed to extract the data and fitted the profiles^32–34^. The *k*^3^-weighted EXAFS spectra were acquired by energy calibration and spectral normalization. For the EXAFS part, the Fourier transformed data in R space of Cu *K*-edge and Co *K*-edge were analyzed by applying a hanning windows (d*k* = 1.0 Å^−1^) to differentiate the EXAFS oscillation from different coordination shells. Subsequently, we performed the least-squares curve parameter fitting to attain the structural parameters around central atoms. The fitted ranges of *k* space were set at 3.4–13.2 Å^−1^ with R range of 1.2–3.0 Å. The four parameters including coordination number (CN), bond length (R), Debye-Waller factor (σ^2^), and *E*_0_ shift (Δ*E*_0_) were fitted without anyone being fixed, constrained, or correlated.

The in situ Cu *K*-edge XAFS measurements were conducted were collected with a home-made XAFS cell. Typically, 8 mg of the catalysts and 40 µL of Nafion were dispersed in 2 mL of ethanol by sonication for 1 h. Then the uniform ink was loaded onto carbon paper with an area of 2 × 2 cm^2^. The mass loading was calculated to be 2 mg cm^−2^. The prepared catalysts, a Ag/AgCl electrode, and a Pt wire were used as the working electrode, reference electrode, and counter electrode, respectively. All electrochemical tests were measured in 1 M KOH electrolyte with 1 M KNO_3_ (45 mL) and controlled by a CHI1140C electrochemical workstation.

### Preparation of the working electrodes

8 mg of the catalysts were dispersed in 2 mL of ethanol by sonication for 1 h. Then 40 µL of Nafion solution was added to the mixture and sonicated for 30 min to obtain a uniform ink. Finally, the uniform ink was loaded onto carbon paper with an area of 2 × 4 cm^2^. The mass loading was calculated to be 1 mg cm^−2^. The area of working electrodes used in the electrochemical measurements was 0.25 cm^2^.

### Electrochemical measurements

The electrochemical measurements were carried out in an H-cell system which was separated by a bipolar membrane with a CHI1140C electrochemical workstation (Chenhua, Shanghai). Ag/AgCl electrode and graphite rod were used as the reference electrode and counter electrode, respectively. For NO_3_^−^ electroreduction, 1 M KOH containing 1 M KNO_3_ solution (60 mL) was evenly distributed to the cathode and anode compartments. The pH value of the electrolyte was determined to be 14 by a FiveEasy Plus pH Meter (METTLER TOLEDO). The applied potentials were measured against the Ag/AgCl reference electrode with 50% *i*R compensation and converted to the RHE reference scale by E (*vs* RHE) = E (*vs* Ag/AgCl) + 0.21 V + 0.0591 × pH – *i*R. The solution resistance was determined to be 4.4 ± 0.2 ohm in the electrolytes by potentiostatic electrochemical impedance spectroscopy at frequencies ranging from 10 Hz to 100 kHz, which was conducted in a standard three-electrode system at ambient conditions. Before the electroreduction test, CV curves were performed until the polarization curves achieved steady-state ones with a scan rate of 10 mV s^−1^. Before the electrolysis, Ar gas was delivered into the cathodic compartment at a rate of 10 mL min^−1^ to remove dissolved O_2_. The LSVs of the catalysts were recorded at a scan rate of 5 mV s^−1^ in 1 M KOH containing 1 M KNO_3_/KNO_2_. The controlled potential electrolysis was performed at applied potentials for 10 min. NO_2_^−^ electroreduction was conducted with the same conditions except that the solution of 1 M KOH containing 1 M KNO_2_ was used as the electrolyte. An absorption cell containing 30 mL of 1 M HCl was set to effectively absorb the possible escaped NH_3_ from the cathode cell. After the electrolysis at each applied potential, the concentration of NH_3_ in the absorption cell was lower than 1 μg mL^−1^. In this case, the volatilization of NH_3_ from the electrolytes could be negligible. Cyclic voltammetric measurements were conducted in a non-faradaic potential window with various scan rates from 50 to 100 mV s^−1^. *C*_dl_ was calculated by plotting the Δ*j* (Δ*j* = *j*_a_ – *j*_c_) at the middle of the corresponding potential window against scan rates. The *j*_a_ and *j*_c_ were the anodic and cathodic current densities, respectively. The slope was twice of *C*_dl_.

### The calculation method for FE

The FE for the product (NH_3_ and NO_2_^−^) was calculated at a given potential as follows:

1 $${{{{{\rm{FE}}}}}}=C\times V\times N\times {{{{{\rm{F}}}}}}/({{{{{\rm{Q}}}}}}\times {{{{{\rm{M}}}}}})$$

*C*: the measured concentration of product (mg mL^−1^),

*V*: the volume of the electrolyte (mL),

*N*: the number of electrons transferred for the product, which is 8 for NH_3_ and 2 for NO_2_^−^,

F: Faraday constant, 96,485 C mol^−1^,

*Q*: total electric charge (C),

M: the relative molecular mass, which is 17 g mol^−1^ for NH_3_ and 46 g mol^−1^ for NO_2_^−^.

### The calculation method for the yield rate of NH_3_ product

The yield rate of NH_3_ product was calculated at a given potential as follows:

2 $$\nu _{{{{{{\rm{NH}}}}}}_{3}}=(C_{{{{{{\rm{NH}}}}}}_{3}}\times V)/(S\times t)\times 60$$

*ν*_NH3_: the yield rate (mg_NH3_ h^−1^ cm^−2^),

*C*_NH3_: the measured concentration of NH_3_ (mg mL^−1^),

*V*: the volume of the electrolyte (mL),

*S*: the area of the catalyst (cm^2^),

*t*: the reduction reaction time (min).

### Determination of ion concentration

Determination of NH_3_ concentration with indophenol blue method^35^. After the electroreduction process, a certain amount of electrolyte was taken out from the electrolytic cell and diluted to the detection range. Then, 2 mL of 1 M NaOH solution containing salicylic acid (5 wt%) and sodium citrate (5 wt%) were added into the aforementioned solution, followed by the addition of 1 mL of 0.05 M NaClO and 0.2 mL of C_5_FeN_6_Na_2_O (1 wt%). After standing in darkness for 2 h, the absorption spectra were measured using a UV-vis spectrophotometer. The concentration of indophenol blue was determined using absorbance at the wavelength of 650 nm. The concentration-absorbance curve was calibrated using standard (NH_4_)_2_SO_4_ solution with a series of concentrations.

Determination of NH_3_ concentration with ^1^H NMR method. After NO_3_^−^ electroreduction, a certain amount of electrolyte was taken out for further quantification by ^1^H NMR (Bruker AVANCE AV III 400). All analyses were performed with 128-time scans. The concentration-integral area curve was calibrated using a standard (NH_4_)_2_SO_4_ solution. Typically, (NH_4_)_2_SO_4_ was dissolved in 20 mL of 1 M KOH electrolyte as a series of standard (NH_4_)_2_SO_4_ solutions with different concentrations. Subsequently, 0.1 mL of dimethyl sulfoxide-*d*_6_ (DMSO-*d*_6_), 0.1 mL of 6 mM 1-propanesulfonic acid 3-(trimethylsilyl) sodium salt (DSS) solution, and 0.08 mL of 6 M HCl to adjust the pH value were added into 0.32 mL of (NH_4_)_2_SO_4_ standard solutions with different concentrations. The signal appeared at 7.23, 7.10, and 6.97 ppm were attributed to NH_4_^+^. The integral areas of the signal of NH_4_^+^ were used to determine the concentration of (NH_4_)_2_SO_4_ compared with the as-known DSS reference.

Determination of NO_3_^−^ concentration^36^. A certain amount of electrolyte was diluted to the detection range of NO_3_^−^. Then, 0.1 mL of 1 M HCl and 0.01 mL of 0.8 wt% sulfamic acid solution were mixed with the diluted electrolyte, followed by shaking for 10 min. Using a UV-vis spectrophotometer, the absorption spectra were collected, obtaining the absorption intensities at a wavelength of 220 and 275 nm. Finally, the calculated absorbance (A = A_220nm_–2A_275nm_) was acquired. The concentration-absorbance curve was calibrated using standard KNO_3_ solutions with a series of concentrations.

Determination of NO_2_^−^ concentration. 4 g of p-aminobenzenesulfonamide, 0.2 g of N-(1-Naphthyl) ethylenediamine dihydrochloride, and 10 mL of phosphoric acid were mixed with 50 mL of water as the color reagent. A certain amount of electrolyte was taken out from the electrolytic cell and diluted to the detection range. 1 mL of H_3_PO_4_ (5 M) was added to the 4 mL of diluted post-electrolysis electrolytes to adjust the pH, followed by the addition of 0.1 mL of color reagent. After standing for 20 min, the absorption spectra were measured using a UV-vis spectrophotometer. The absorption intensity at a wavelength of 540 nm was recorded. The concentration-absorbance curve was calibrated using standard KNO_2_ solution with a series of concentrations.

### Determination of other liquid and gaseous product

The amount of NH_2_OH was determined by ^1^H NMR after NH_2_OH was captured by excess amount of C_2_H_2_O_3_ through oximation process. Specifically, 0.4 mL of the electrolyte after the NO_3_^−^ electroreduction was mixed with 10 μL of 50% C_2_H_2_O_3_ solution, followed by the addition of 0.1 mL of DMSO-*d*_6_ (99%) and 0.1 mL of 6 mM DSS solution. The integral area of the signal appeared at 7.46 ppm were used to determine the concentration of NH_2_OH compared with the as-known DSS reference. In this work, the amount of NH_2_OH was below the detection limit for both NO_3_^−^ and NO_2_^−^ reduction over Co_3_O_4_/Cu_1_-N-C.

Nitrogen oxides including NO, NO_2_, and N_2_O have been detected by an infrared gas analyzer (THA100S). H_2_ and N_2_ have been detected by an on-line gas chromatograph (GC-2014) equipped with a flame ionization detector and a thermal conductivity detector.

The FE for gaseous products were calculated by the following equation:

3 $${{{{{\rm{FE}}}}}}=x\times {V}_{{gas}}\times N\times F/(Q\times {V}_{m})$$

*x*: the measured mole fraction of product,

*V*_gas_: the total volume of the gas (L),

*N*: the number of electrons transferred for the product, which is 3 for NO, 8 for N_2_O, 2 for H_2_ and 10 for N_2_,

F: Faraday constant, 96485 C mol^−1^,

*Q*: total electric charge (C),

*V*_m_: the molar volume of the gas, 24.5 L mol^−1^.

### Isotope labeling experiments

The isotopic labeling experiment used K^15^NO_3_ with ^15^N enrichment of 99% as the feeding N-source to clarify the source of ammonia. 1 M KOH was used as the electrolyte and K^15^NO_3_ with a concentration of 1 M was added into the cathode compartment as the reactant. After the electrolysis, 0.1 mL of DMSO-*d*_*6*_ and 0.1 mL of 6 mM DSS solution were added into 0.4 mL of the electrolyte, followed by adding 0.05 mL of HCl (0.1 M) to adjust the pH of the solutions. Then the obtained ^15^NH_4_^+^ was identified on a Varian 400 MHz NMR spectrometer (Bruker AVANCE AV III 400).

### Kinetic evaluation

The electrolysis at −1.0 V *vs* RHE were conducted for different time to acquire the rate constant in 1 M KOH containing 1 M NO_3_^−^ or 1 M NO_2_^−^. The reaction constant (*k*_1_ for NO_3_^−^ reduction and *k*_2_ for NO_2_^−^ reduction) was calculated by plotting the concentration of NO_3_^−^ or NO_2_^−^ against the time of reaction, supposing that the concentrations of NO_3_^−^ or NO_2_^−^ declined exponentially as per first-order rate.

4 $${C}_{t}={C}_{0\exp }({-}k\times t)$$

*C*_0_: initial concentration of NO_3_^−^ or NO_2_^−^ (g mL^−1^),

*C*_t_: the concentration of NO_3_^−^ or NO_2_^−^ at time *t* (g mL^−1^),

*t*: the time of reaction (min).

### Adsorption experiments

To determine the adsorption capacities of Co_3_O_4_/Cu_1_-N-C, Cu_1_-N-C, and Co_3_O_4_/N-C, 5 mg of catalysts were added to each 25 mL of NO_3_^−^ or NO_2_^−^ solutions with the initial concentration of 1 M under stirring for 2 h, respectively. The solutions were separated by filtration using the 0.22 μm microporous membrane filter. For high concentration of NO_3_^−^ or NO_2_^−^, the solution was diluted before absorbance measurements. The adsorption capacity was calculated using the following equation:

5 $${q}_{e}=({C}_{0}{-}{C}_{e}) \times V/m$$

q_e_: the adsorption capacity (g g_cat._^−1^),

*C*_0_: the initial concentration of NO_3_^−^ or NO_2_^−^ (g mL^−1^),

*C*_e_: the measured concentration of NO_3_^−^ or NO_2_^−^ after the adsorption (g mL^−1^),

*V*: the volume of the electrolyte (mL),

*m*: the mass of the catalyst (g).

### In situ FTIR measurements

Using a Nicolet iS50 FTIR spectrometer (Thermo Scientific) with a built-in MCT detector, we obtained the in situ electrochemical FTIR spectra. Typically, 2 mg of catalysts and 20 µL of Nafion dispersed in 2 mL of ethanol were sonicated for 1 h. Then the mixture was loaded onto the Au-coated Si prism to completely cover the Au film. The prepared prism was used as the working electrode after being dried naturally. The reference electrode and counter electrode was a Ag/AgCl electrode and a Pt wire, respectively. The photograph of the in situ FTIR electrochemical cell was shown in Supplementary Fig. 36. All electrochemical tests were measured in 1 M KOH electrolyte with 0.1 M KNO_3_ (30 mL) and controlled by a CHI1140C electrochemical workstation. All experiments were conducted at room temperature. The background spectra of the working electrode were obtained at an open-circuit potential before the electrochemical tests. All of the spectra were collected in absorbance by averaging 32 scans at a resolution of 4 cm^−1^.

### In situ Raman measurements

In situ Raman was carried out using Lab RAM HR Evolution (Horiba) equipped with a 50× microscope objective. The excitation wavelength was 532 nm with 10% intensity. The photograph of electrochemical cell for in situ Raman measurement was shown in Supplementary Fig. 37. Typically, 2 mg of catalysts and 20 µL of Nafion were dispersed in 2 mL of ethanol by sonication for 1 h. Then the electrocatalysts were deposited on fluorine tin oxide-coated glass as the working electrode. A Ag/AgCl electrode, and a Pt wire were used as the reference electrode, and counter electrode, respectively. All electrochemical tests were measured in 1 M KOH electrolyte with 1 M KNO_3_/KNO_2_ (5 mL) and controlled by a CHI1140C electrochemical workstation. All experiments were conducted at room temperature. Each spectrum was collected by integration twice, 60 s per integration. To determine the local concentration of NO_2_^−^ near the surface of catalysts, we employed an internal standard method during the in situ Raman measurements.

### DFT calculations

DFT calculations were performed using the Vienna Ab-Initio Simulation Package (VASP) code at the GGA level within the PAW-PBE formalism^37^. DFT-D3 method with Becke-Jonson damping is performed for the van der Waals correction. The three-layer Co_3_O_4_ (100) slab model was adopted with a vacuum of 15 Å. The total energy calculations were performed using a 2 × 2 × 1 grid and a plane wave cut-off energy of 400 eV. Atoms in the bottom two layers were fixed. A U value of 3.5 eV was applied to the 3*d* states of Co to describe the strong on-site Coulomb interactions due to the localization of the Co 3*d* states^38^. For the model of CuN_4_ (no atoms were fixed), the total energy calculations were performed using a 3 × 3 × 1 grid and a plane wave cut-off energy of 400 eV. All atoms, which were not fixed, including adsorbates were allowed to relax until the force on each ion was smaller than 0.02 eV/Å.

We calculated the Gibbs free energy (*G*) for each species as follows:

6 $$G={E}_{{DFT}}+{E}_{{ZPE}}{-}{TS}$$

where *E*_*DFT*_, *E*_*ZPE*_, and *TS* represent the DFT-optimized total energy, zero point energy (ZPE), and entropy contribution, respectively (T is the temperature, 298.15 K). It is assumed that S = 0 for all the adsorbed species.

### Supplementary information

Supplementary Information

Peer Review File

### Source data

Source data

## Data Availability

The data that support the findings of this study are available from the corresponding author upon request. The source data underlying Figs. 1–4 and Supplementary Figs. 1–44 are provided as a Source Data file. Source data are provided with this paper.
