# Supplementary material for: Efficient tandem electroreduction of nitrate into ammonia through coupling Cu single atoms with adjacent Co3O4
Source: Nat Commun. 2024 Apr 29;15:3619. doi: 10.1038/s41467-024-48035-4 (PMC11059385; doi:10.1038/s41467-024-48035-4)
Supplement: Supplementary file 1 — Supplementary Information [file 41467_2024_48035_MOESM1_ESM.pdf]

Supplementary Information for

Efficient tandem electroreduction of nitrate into ammonia through coupling Cu single atoms with adjacent Co_3O_4

Yan Liu[1,7], Jie Wei[1,7], Zhengwu Yang[1,7], Lirong Zheng[2,], Jiankang Zhao[1,], Zhimin Song[1,], Yuhan Zhou[1,], Jiajie Cheng[3,], Junyang Meng[1,], Zhigang Geng[1]*, Jie Zeng[1,4,5,6,]*

[1]Hefei National Research Center for Physical Sciences at the Microscale, University of Science and Technology of China, Hefei, Anhui 230026, P. R. China

[2]Institute of High Energy Physics, Chinese Academy of Sciences, Beijing 100049, P. R. China

[3]Department of Physics, University of Science and Technology of China, Hefei, Anhui 230026, P. R. China

[4]CAS Key Laboratory of Strongly-Coupled Quantum Matter Physics, University of Science and Technology of China, Hefei, Anhui 230026, P. R. China

[5]Key Laboratory of Surface and Interface Chemistry and Energy Catalysis of Anhui Higher Education Institutes, Department of Chemical Physics, University of Science and Technology of China, Hefei, Anhui 230026, P. R. China

[6]School of Chemistry & Chemical Engineering, Anhui University of Technology, Ma'anshan, Anhui 243002, P. R. China

[7]These authors contributed equally to this work.

[*]Corresponding author. E-mail: gengzg@ustc.edu.cn (Z.G.); E-mail: zengj@ustc.edu.cn (J.Z.).

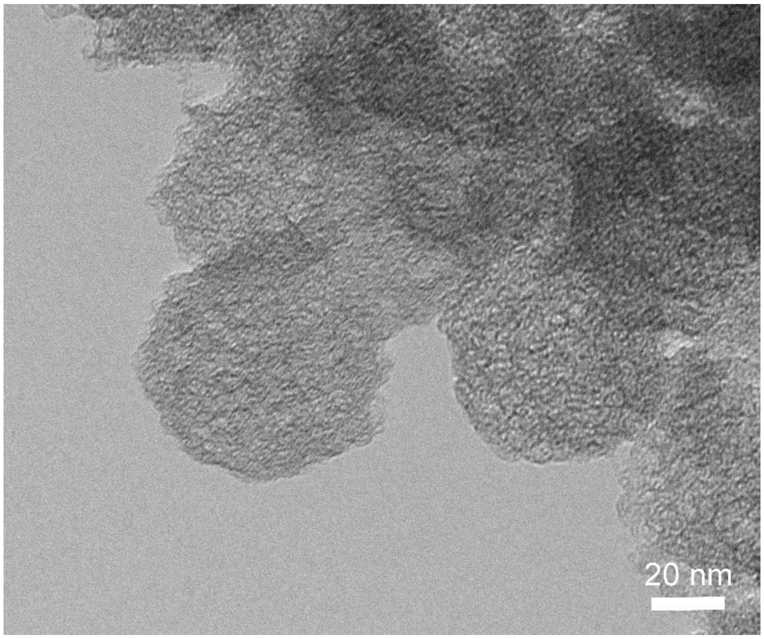

Supplementary Fig. 1. TEM image of Cu_1-N-C. Cu_1-N-C exhibited the amorphous carbon structure without any observable metallic particles.

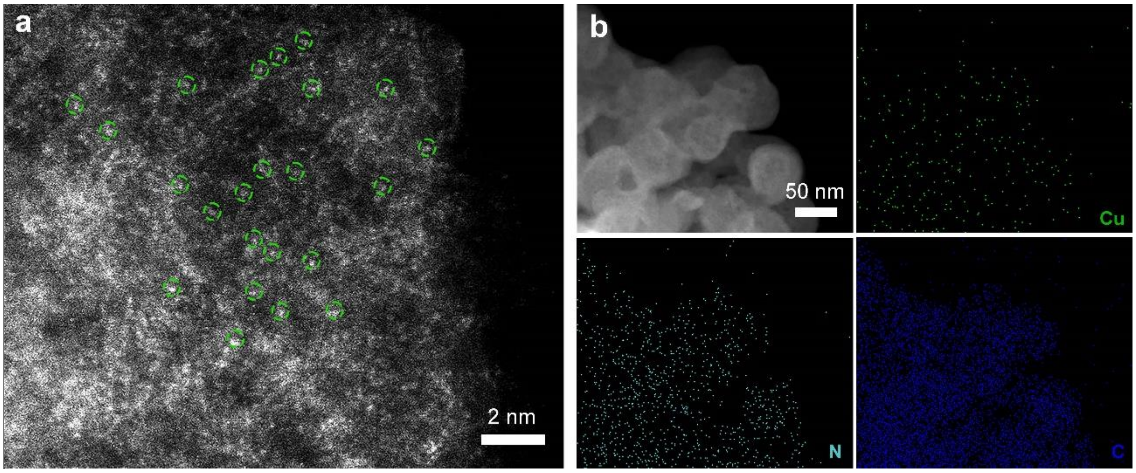

Supplementary Fig. 2. Characterization of Cu_1-N-C. (**a**) Aberration-corrected HAADF-STEM image and (**b**) EDS elemental mapping results of Cu_1-N-C. These results demonstrate the atomically dispersed Cu in Cu_1-N-C.

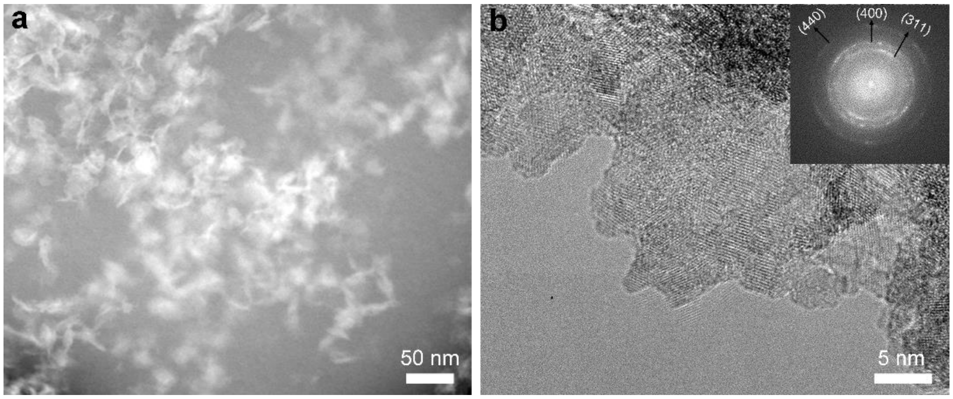

Supplementary Fig. 3. Characterization of Co_3O_4/Cu_1-N-C. (**a**) HAADF-STEM image of Co_3O_4/Cu_1-N-C and (**b**) HRTEM image and the corresponding SAED pattern (the inset) of Co_3O_4/Cu_1-N-C. The lattice fringe distances were well indexed to (311), (400), and (440) planes of Co_3O_4, confirming the successful formation of Co_3O_4.

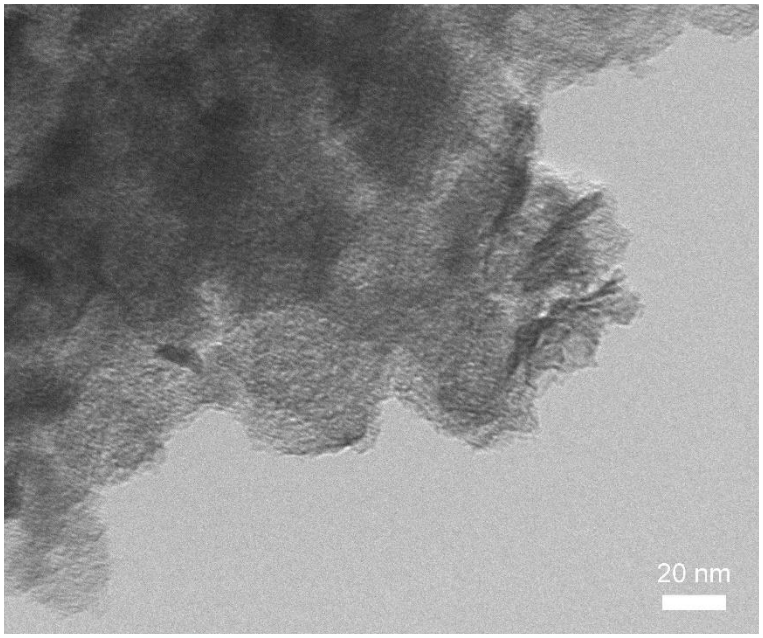

Supplementary Fig. 4. TEM image of Co_3O_4/N-C.

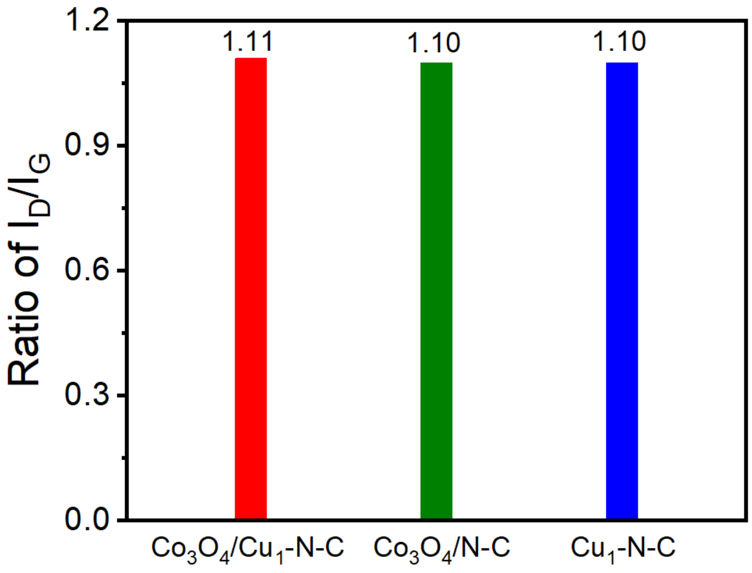

Supplementary Fig. 5. **The ratio of I_D/I_G for Co_3O_4/Cu_1-N-C, Co_3O_4/N-C and Cu_1-N-C.**

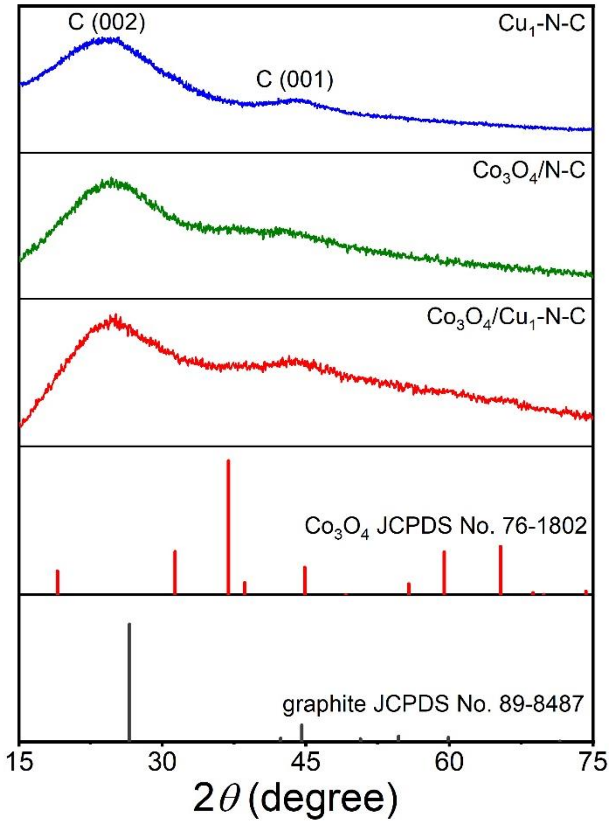

Supplementary Fig. 6. XRD patterns of Cu_1-N-C, Co_3O_4/N-C, and Co_3O_4/Cu_1-N-C. The three samples all exhibited two broad peaks at 24.5° and 44.0°, which were attributed to the (002) and (001) facets of graphite carbon.

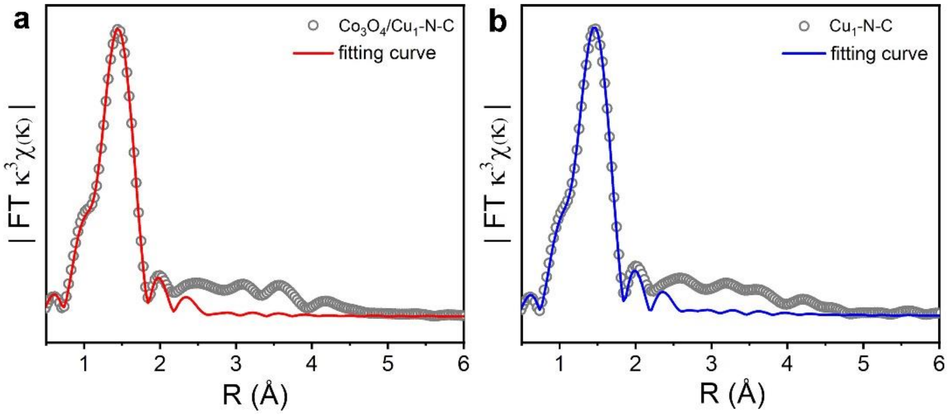

Supplementary Fig. 7. Cu *K*-edge EXAFS fitting curves of (a) Co_3O_4/Cu_1-N-C and (b) Cu_1-N-C.

Supplementary Table 1. EXAFS data fitting results of Co_3O_4/Cu_1-N-C and Cu_1-N-C.

Sample	Shell	*CN*	R (Å)	σ^2	ΔE_0 (eV)
Co_3O_4/Cu_1-N-C	Cu-N	3.7 ± 0.7	1.93 ± 0.03	0.007	1.3
Cu_1-N-C	Cu-N	3.9 ± 0.7	1.93 ± 0.03	0.007	0.4

CN represents coordination number, R represents bond distance, σ^2 represents Debye-Waller factor, and ΔE_0 represents edge-energy shift.

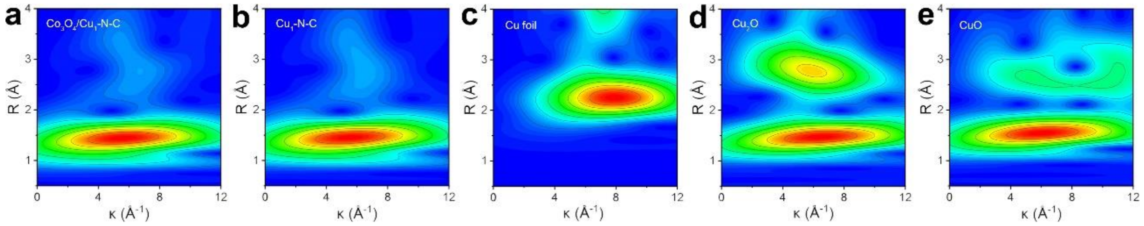

Supplementary Fig. 8. Wavelet transformed EXAFS spectra of (a) Co_3O_4/Cu_1-N-C, (b) Cu_1-N-C, (c) Cu foil, (d) Cu_2O and (e) CuO. The WT-EXAFS spectra of Co_3O_4/Cu_1-N-C and Cu_1-N-C both showed the maximum intensity at around 5.6 $Å^{-1}$, demonstrating the Cu-N coordination structure in the two catalysts.

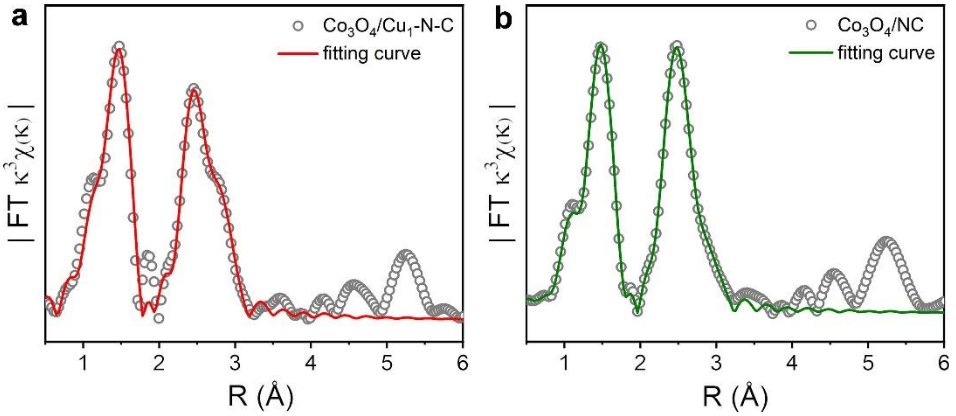

Supplementary Fig. 9. Co *K*-edge EXAFS fitting curves of (a) Co_3O_4/Cu_1-N-C and (b) Co_3O_4/N-C. The coordination structure of Co_3O_4 species were similar on the Cu_1-N-C and N-doped carbon supports.

Supplementary Table 2. EXAFS data fitting results of Co *K*-edge for Co_3O_4/Cu_1-N-C and Cu_1-N-C.

Sample	Shell	*CN*	R (Å)	σ^2	ΔE_0 (eV)
Co_3O_4/Cu_1-N-C	Co-O	4.0 ± 0.8	1.89 ± 0.03	0.007	-4.7
	Co-O-Co	2.0 ± 0.4	2.86 ± 0.03	0.005	-2.1
	Co-O-Co	1.9 ± 0.4	3.09 ± 0.03	0.008	-2.1
Co_3O_4/N-C	Co-O	4.2 ± 0.8	1.89 ± 0.03	0.004	-4.2
	Co-O-Co	2.6 ± 0.5	2.84 ± 0.03	0.005	1.2
	Co-O-Co	0.6 ± 0.1	3.16 ± 0.03	0.005	1.2

CN represents coordination number, R represents bond distance, σ^2 represents Debye-Waller factor, and ΔE_0 represents edge-energy shift.

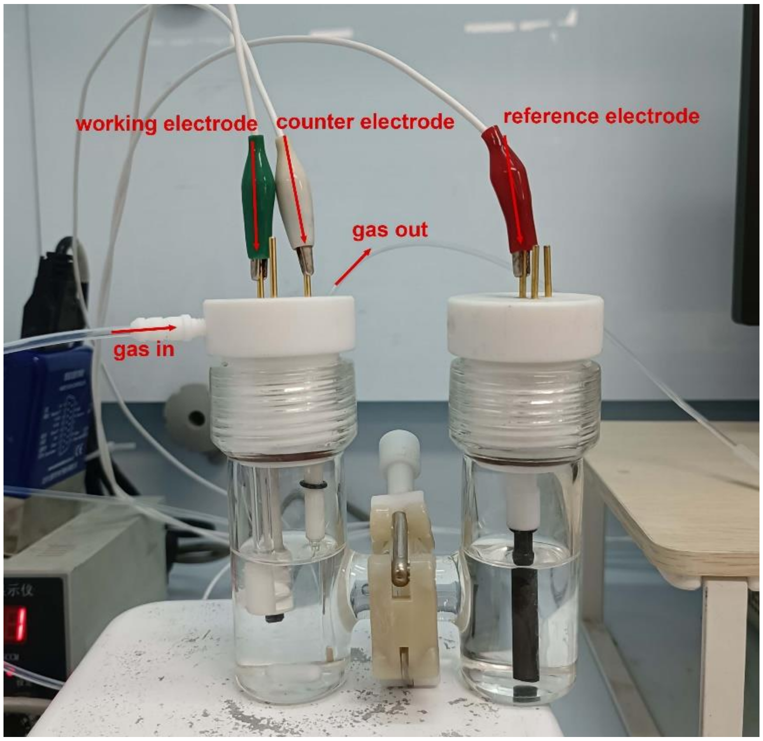

Supplementary Fig. 10. The photograph of the H-cell during the electrochemical measurements.

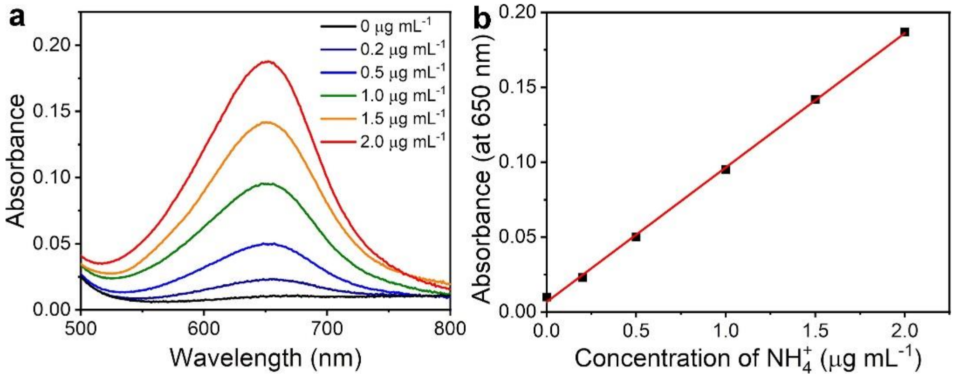

Supplementary Fig. 11. Determination of NH_4^+. (**a**) UV-vis curves and (**b**) concentration-absorbance curve of NH_4^+ solution with a series of standard concentrations. The standard curve showed linear relation of absorbance with NH_4^+ concentration (y = 0.089x + 0.006, R^2 = 0.9992).

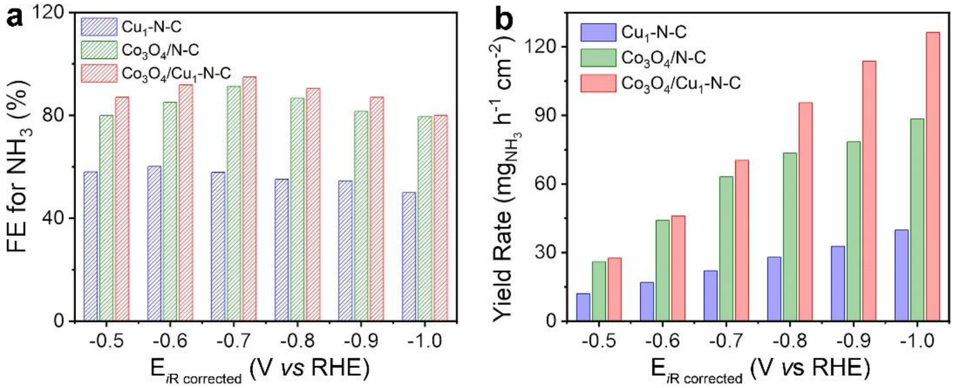

Supplementary Fig. 12. Catalytic performance toward NO_2^- electroreduction. (**a**) FE for NH_3 and (**b**) yield rate for NH_3 of Cu_1-N-C, Co_3O_4/N-C, and Co_3O_4/Cu_1-N-C at different applied potentials in 1 M NO_2^-.

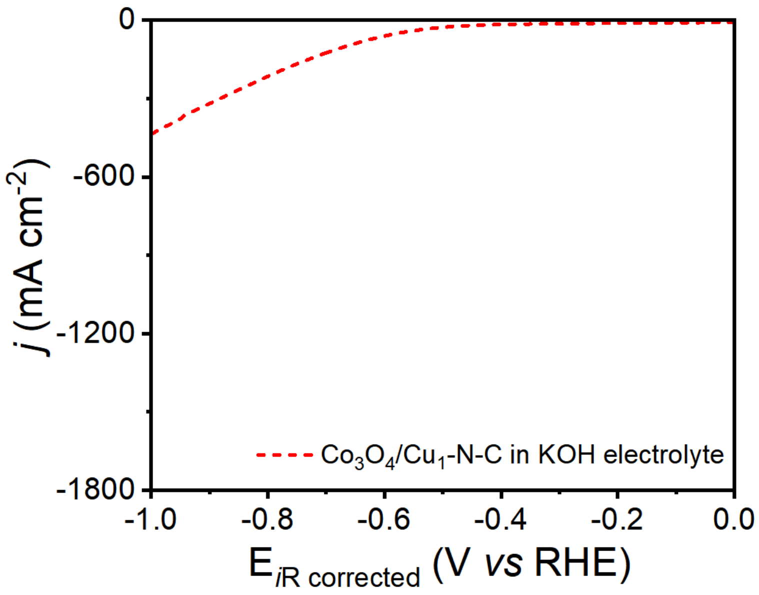

Supplementary Fig. 13. LSV curve of Co_3O_4/Cu_1-N-C in 1 M KOH electrolyte without NO_3^-.

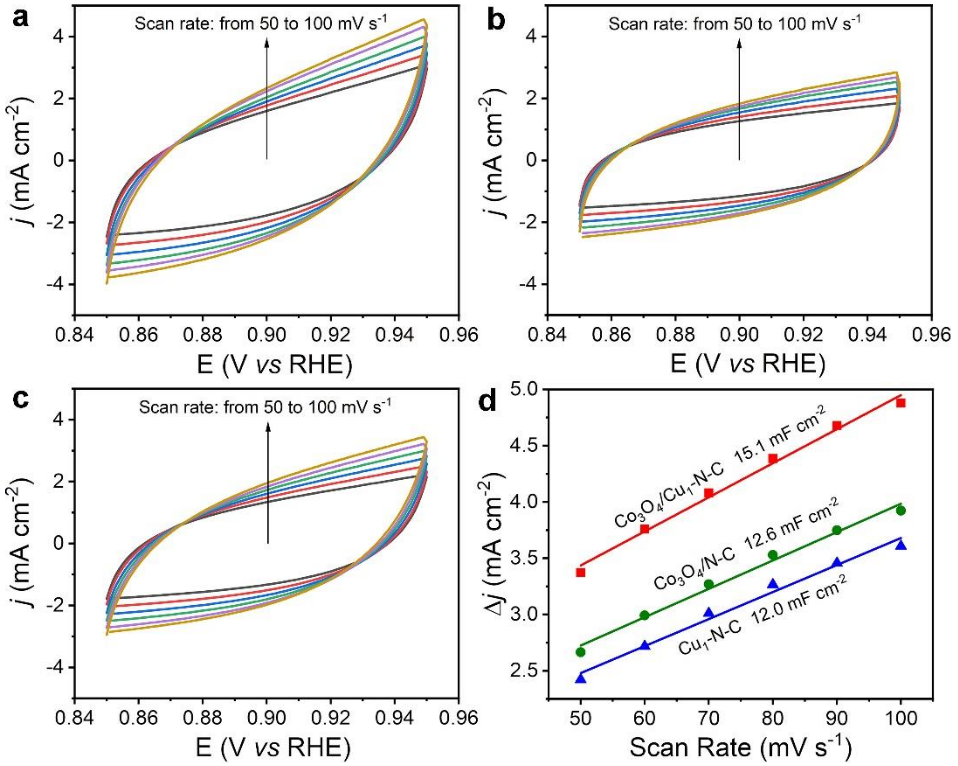

Supplementary Fig. 14. CV curves and charging current density differences of Co_3O_4/Cu_1-N-C, Cu_1-N-C, and Co_3O_4/N-C. CV curves for (**a**) Co_3O_4/Cu_1-N-C, (**b**) Cu_1-N-C, and (**c**) Co_3O_4/N-C at different scan rates from 50 to 100 mV s^{-1}, respectively. (**d**) Charging current density differences plotted against scan rates of Co_3O_4/Cu_1-N-C, Cu_1-N-C, and Co_3O_4/N-C.

Supplementary Table 3. The comparison of catalytic performance over recently reported electrocatalysts toward NO_3^- electroreduction.

Catalysts	Mass loading ($mg\ cm^{-2}$)	Potential (V vs RHE)	Electrolyte	Stability	Electrode type	FE (%)	Yield rate ($mg_{NH_3}\ h^{-1}\ cm^{-2}$)	Ref.
$Cu_{50}Ni_{50}$	/	-0.15	0.1 M NO_3^- + 0.1 M KOH	12 h	Glassy carbon	99.0	/	1
CuCoSP	/	-0.175	0.1 M NO_3^- + 0.1 M KOH	10 h	Cu foil	~90.6	19.9	2
Rh@Cu-0.6%	/	-0.2	0.1 M NO_3^- + 0.05 M Na_2SO_4	14 h	Cu foil	93.0	21.6	3
Cu-N-C SAC	1.33	-1.0	0.1 M NO_3^- + 0.1 M KOH	20 cycles	Carbon paper	84.7	4.5	4
CoO_x	1.75	-0.3	0.1 M NO_3^- + 0.1 M KOH	/	Carbon cloth	93.4	2.9	5
Ru-CuNW	/	-0.135	0.1 M NO_3^- + 1 M KOH	/	Cu foam	95.6	76.5	6
Ni_{35}/NC-sd	2	-0.5	0.3 M NO_3^- + 0.5 M Na_2SO_4	5 cycles	Ti mesh	99	5.1	7
Ti foil	/	-1.0	0.3 M NO_3^- + 0.1 M HNO_3	8 h	Ti foil	82	/	8
MWCNTs	0.4	-0.85	0.4 M NO_3^- + 0.1 M PBS	/	Carbon paper	73	/	9
Fe SAC	0.4	-0.85	0.5 M NO_3^- + 0.1 M Na_2SO_4	20 cycles	Glassy carbon	74.9	7.8	10
CoP NAs/CFC	0.1143	-0.5	1 M NO_3^- + 1 M NaOH	20 h	Glassy carbon	~100	16.3	11
Ru-ST-12	0.185	-1.0	1 M NO_3^- + 1 M KOH	100 h	Carbon paper	~100	19.9	12
CNS-CoP	/	-1.03	1 M NO_3^- + 1 M KOH	123 h	Carbon cloth	90.5	55.7	13
Ir nanotubes	/	0.06	1 M NO_3^- + 0.1 M $HClO_4$	/	Glassy carbon	84.7	/	14

Bi-Clred	0.51	-0.8	1 M NO_3^- + 1 M KOH	6 cycles	Glassy carbon	~75	~28	[15]
Cu/CuAu	0.2	-0.6	1 M NO_3^- + 1 M KOH	20 cycles	Carbon paper	85.5	1.7	[16]
Co_3O_4/Cu NC	1	-1.0	1 M NO_3^- + 1 M KOH	20 cycles	Carbon paper	97.7	114.0	This work

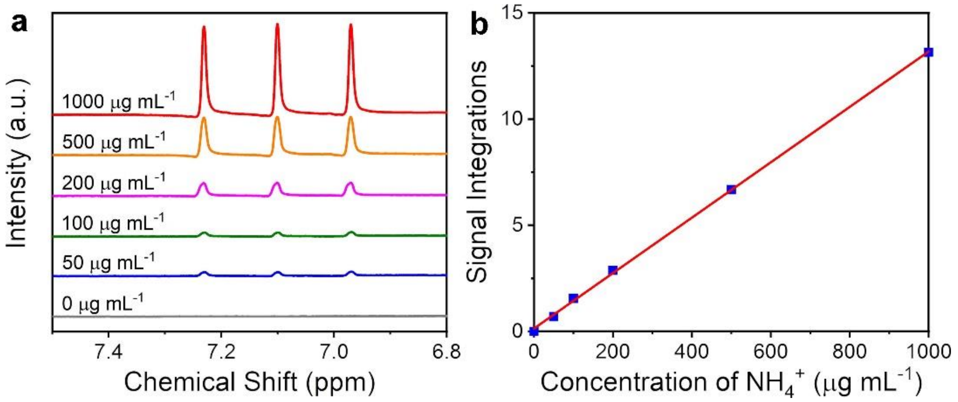

Supplementary Fig. 15. Determination of NH_4^+. (**a**) ^1H NMR spectra of standard $(NH_4)_2SO_4$ solutions with a series of standard concentrations, respectively. (**b**) Concentration-integral area curve for the standard $(NH_4)_2SO_4$ solutions. The standard curve showed linear relation of signal integration with NH_4^+ ion concentration ($y = 0.013x + 0.137$, $R^2 = 0.9994$).

Supplementary Table 4. The yield rate of NH_3 (mg_{NH3} h^{-1} cm^{-2}) over Co_3O_4/Cu_1-N-C determined by indophenol blue method and ^{1}H NMR method, respectively.

The applied potential	Indophenol blue method	^{1}H NMR
-0.5 V *vs* RHE	36.7	37.0
-0.6 V *vs* RHE	50.9	49.9
-0.7 V *vs* RHE	65.7	66.4
-0.8 V *vs* RHE	91.7	89.6
-0.9 V *vs* RHE	109.4	106.9
-1.0 V *vs* RHE	114.0	112.7

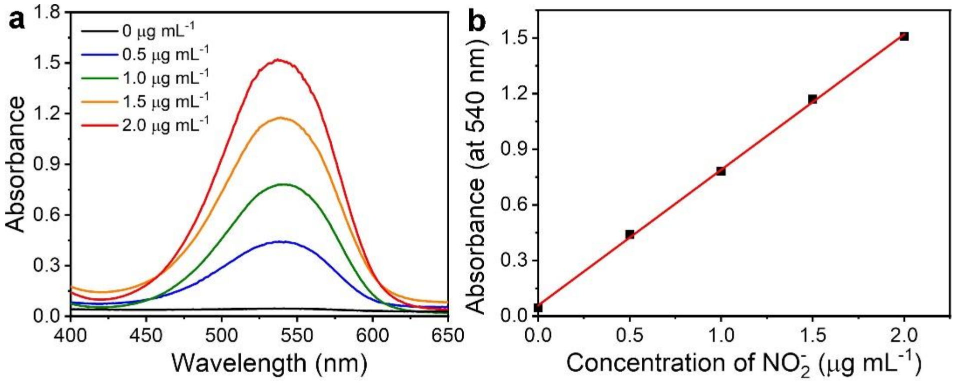

Supplementary Fig. 16. Determination of NO_2^-. (**a**) UV-vis curves and (**b**) concentration-absorbance curve of NO_2^- solution with a series of standard concentrations. The standard curve showed linear relation of absorbance with NO_2^- concentration ($y = 0.731x + 0.058$, $R^2 = 0.9991$).

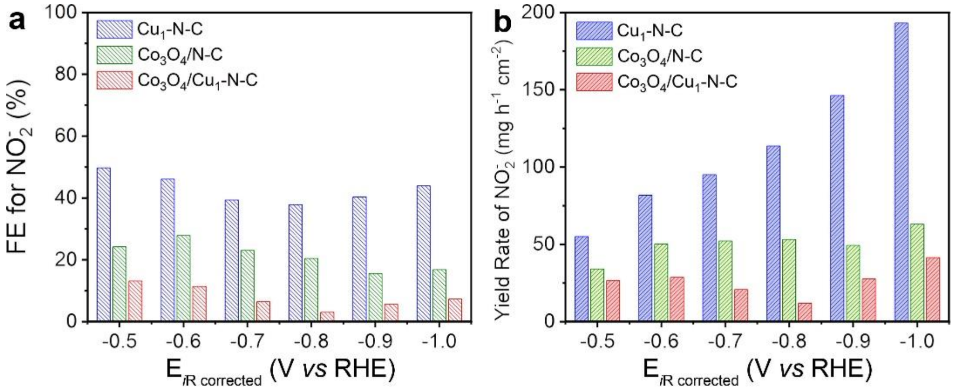

Supplementary Fig. 17. Catalytic performance for NO_2^- production. (**a**) FE for NO_2^- and (**b**) yield rate of NO_2^- over Cu_1-N-C, Co_3O_4/N-C, and Co_3O_4/Cu_1-N-C at different applied potentials in 1 M NO_3^-, respectively. Among the three catalysts, Cu_1-N-C exhibited the highest FE for NO_2^-, indicating the excessive accumulation of NO_2^- during the electroreduction of NO_3^-, which hampered the formation of NH_3 for Cu_1-N-C. Notably, FE for NO_2^- of Co_3O_4/Cu_1-N-C was much lower than that of Cu_1-N-C, suggesting that the composition of Co_3O_4 with Cu_1-N-C promoted the subsequent conversion of NO_2^-.

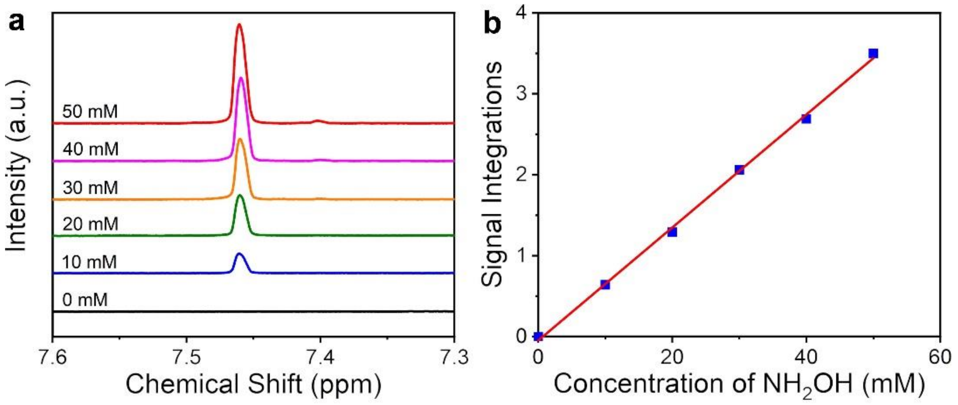

Supplementary Fig. 18. Determination of NH_2OH. (**a**) ^{1}H NMR spectra of standard NH_2OH solutions with a series of standard concentrations, respectively. (**b**) Concentration-integral area curve for the standard NH_2OH solutions. The standard curve showed linear relation of signal integration with NH_2OH concentration ($y = 0.070x - 0.048$, $R^2 = 0.9982$).

Supplementary Table 5. The FE of other products over Co_3O_4/Cu_1-N-C toward NO_3^- and NO_2^- electroreduction at -0.8 V *vs* RHE, respectively.

	NO_3^- electroreduction	NO_2^- electroreduction
NH_3	97.7%	94.9%
NH_2OH	/	/
NO	0.6%	1.3%
NO_2	/	/
N_2O	0.5%	0.8%
N_2	2.0%	7.3%
H_2	0.6%	0.3%

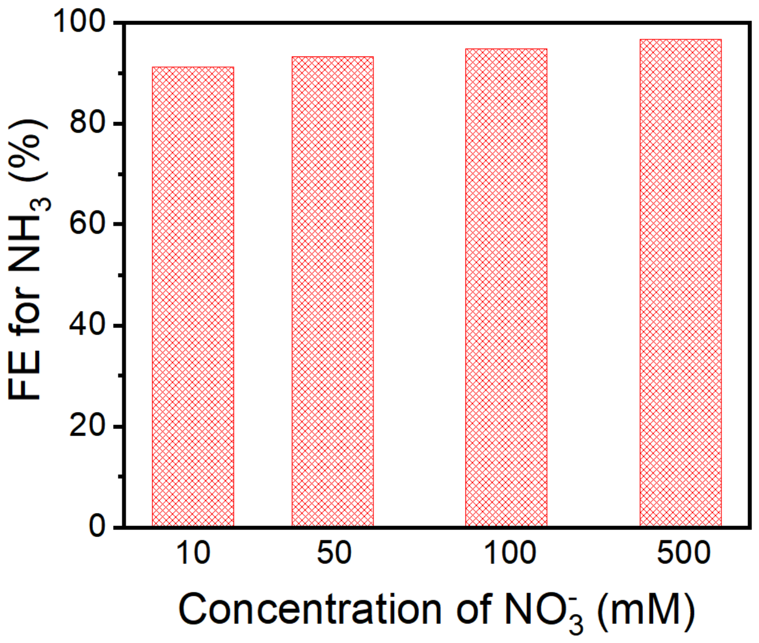

Supplementary Fig. 19. The FE for NH_3 over Co_3O_4/Cu_1-N-C at different concentrations of NO_3^- ranging from 10 mM to 500 mM.

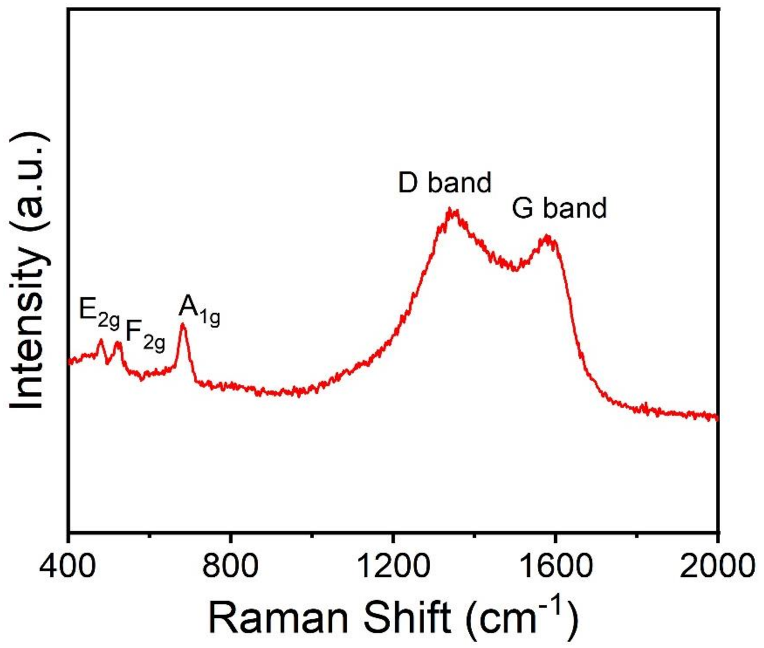

Supplementary Fig. 20. Raman spectrum for Co_3O_4/Cu_1-N-C after the electrolysis.

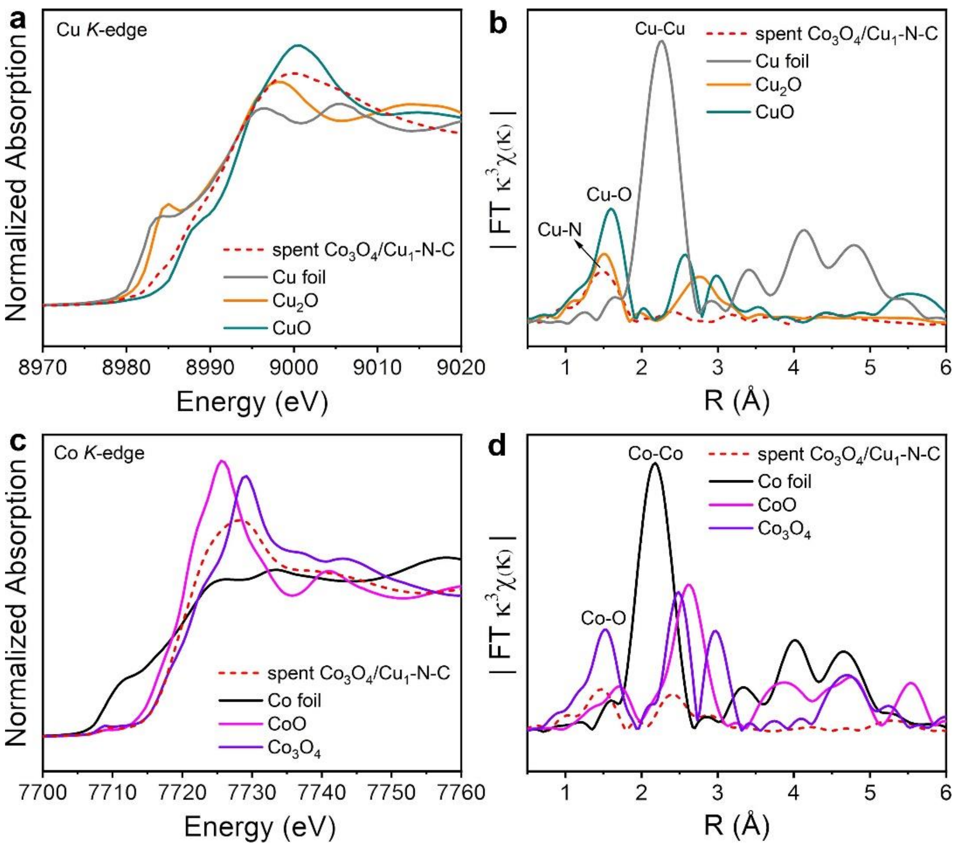

Supplementary Fig. 21. Characterization of Co_3O_4/Cu_1-N-C after the electrolysis. Cu *K*-edge (**a**) XANES spectra and (**b**) EXAFS spectra for Co_3O_4/Cu_1-N-C after the electrolysis, Cu foil, Cu_2O, and CuO. Co *K*-edge (**c**) XANES spectra and (**d**) EXAFS spectra for Co_3O_4/Cu_1-N-C after the electrolysis, Co foil, CoO, and Co_3O_4.

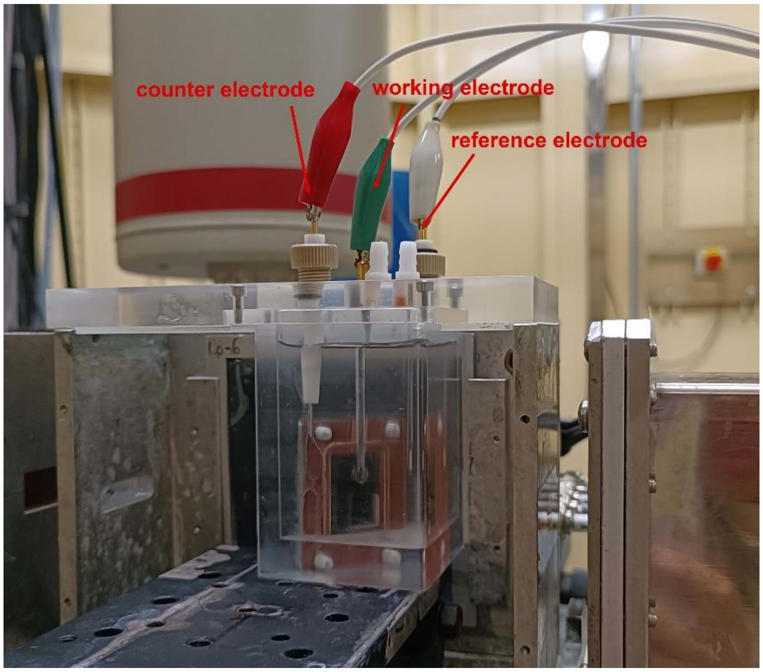

Supplementary Fig. 22. A photograph of the homemade in situ XAFS cell during the operation.

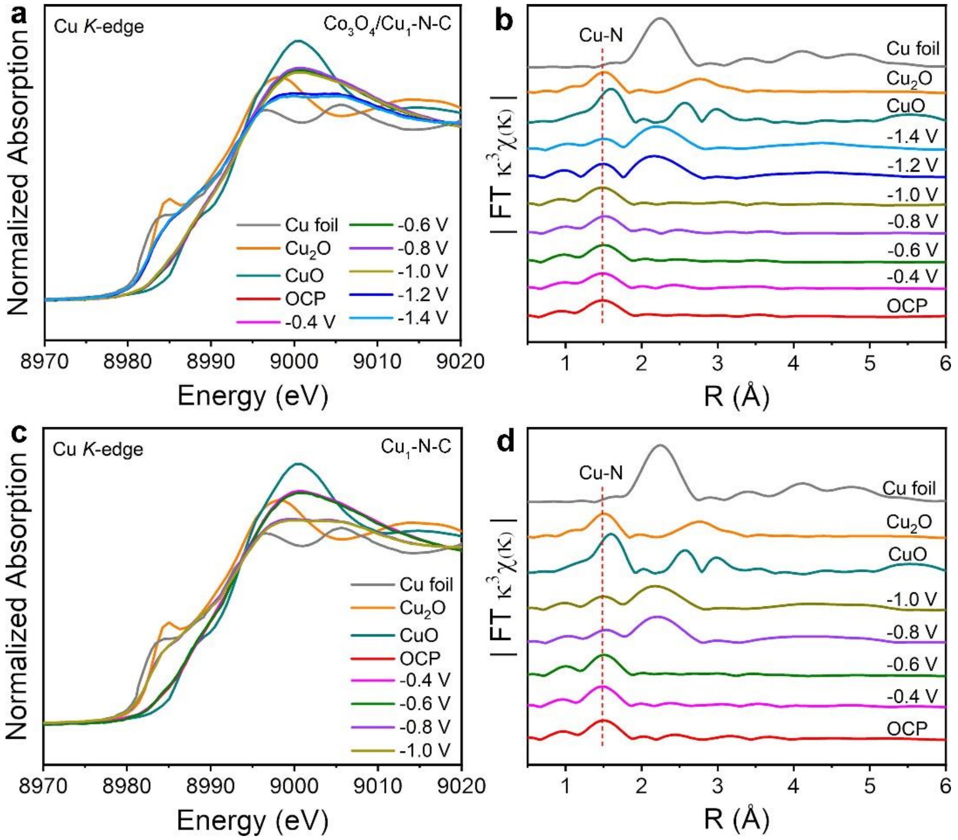

Supplementary Fig. 23. ***In situ*** **Cu** ***K*****-edge (a) XANES spectra and (b) EXAFS spectra for** $\mathbf{Co_3O_4/Cu_1}$**-N-C.** ***In situ*** **Cu** ***K*****-edge (c) XANES spectra and (d) EXAFS spectra for** $\mathbf{Cu_1}$**-N-C.** As the potentials increased from -0.4 V to -1.0 V *vs* RHE, the edge profiles of *in situ* XANES spectra for Co_3O_4/Cu_1-N-C showed no discernible change (Supplementary Fig. 23a). Moreover, no Cu-Cu bond was observed in the EXAFS spectra, demonstrating that Cu single atoms remained in the atomically dispersed state under the potentials of electrochemical tests (Supplementary Fig. 23b). However, when the potentials further increased, a prominent decline in the edge energy of XANES spectra was observed and the peak of Cu-Cu bond appeared in the EXAFS spectra for Co_3O_4/Cu_1-N-C. These phenomena manifested that the Cu atoms aggregated into clusters under the potential as high as -1.2 V *vs* RHE. With regard to Cu_1-N-C, the decrease in the edge energy of XANES spectra and the emergence of Cu-Cu bond were both discerned at -0.8 V *vs* RHE (Supplementary Figs. 23c and d). The introduction of Co_3O_4 nanosheets partly prevented the aggregation of Cu atoms, which might be attributed to the segregation of Cu atoms in Cu_1-N-C by Co_3O_4 nanosheets as the fence, requiring higher energy to drive the separated Cu atom in Co_3O_4/Cu_1-N-C to aggregate into particles.

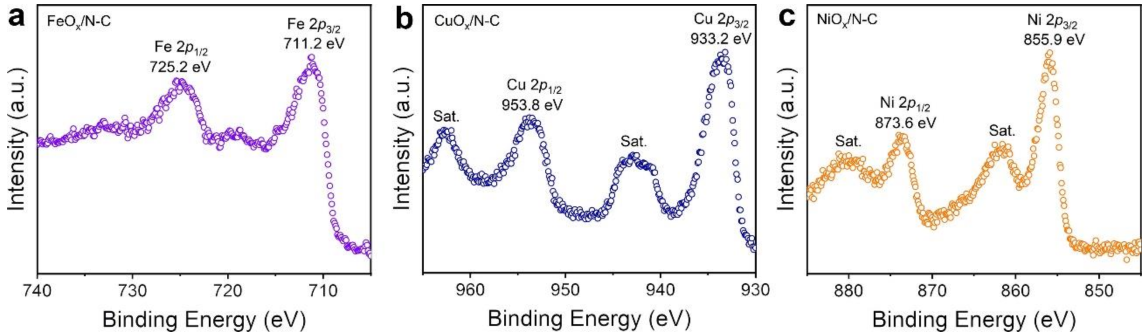

Supplementary Fig. 24. (a) Fe 2*p* XPS spectra of FeO_x/N-C, (b) Cu 2*p* XPS spectra of CuO_x/N-C, and (c) Ni 2*p* XPS spectra of NiO_x/N-C. The Fe 2*p* XPS spectra of FeO_x/N-C exhibited two signals at 725.2 and 711.2 eV, which were attributed to Fe^{3+} $2p_{1/2}$ and Fe^{3+} $2p_{3/2}$, respectively. The Cu 2*p* XPS spectra of CuO_x/N-C showed two peaks at 953.8 and 933.2 eV, which were assigned to Cu^{2+} $2p_{1/2}$ and Cu^{2+} $2p_{3/2}$, respectively. The Ni 2*p* XPS spectra of NiO_x/N-C displayed two signals at 837.6 and 855.9 eV, which were assigned to Ni^{2+} $2p_{1/2}$ and Ni^{2+} $2p_{3/2}$, respectively.

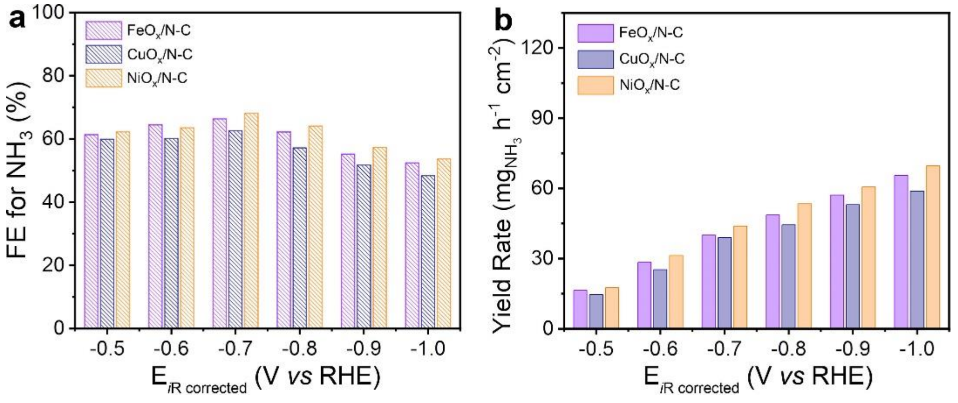

Supplementary Fig. 25. Catalytic performance of FeO_x/N-C, CuO_x/N-C, and NiO_x/N-C. (a) FE for NH_3 and (**b**) yield rate for NH_3 of FeO_x/N-C, CuO_x/N-C, and NiO_x/N-C at different applied potentials with 1 M NO_2^-.

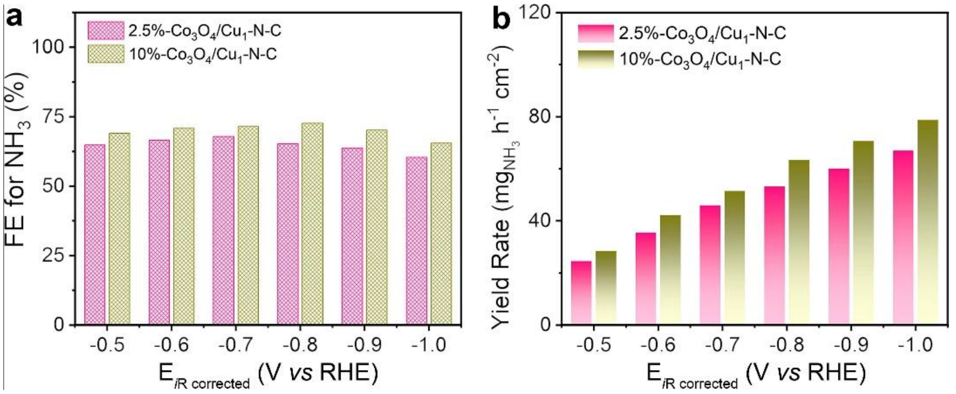

Supplementary Fig. 26. Catalytic performance of Co_3O_4/Cu_1-N-C with different loading of Co_3O_4. (**a**) FE for NH_3 and (**b**) yield rate of NH_3 over Co_3O_4/Cu_1-N-C prepared by controlling the amount of Co precursor as 22.5 and 67.5 mg, respectively. The corresponding samples were denoted as 2.5%-Co_3O_4/Cu_1-N-C and 10%-Co_3O_4/Cu_1-N-C, respectively. The insufficient loading of Co_3O_4 on Cu_1-N-C could not ensure the efficient conversion of the accumulated NO_2^-, leading to undesirable performance. However, the excess amount of Co_3O_4 would completely cover the surface of Cu_1-N-C, impeding the fully exposure of Cu sites to adsorb NO_3^-. As such, the moderate ratio between Cu single atom and Co_3O_4 are beneficial to the performance of NO_3^- electroreduction.

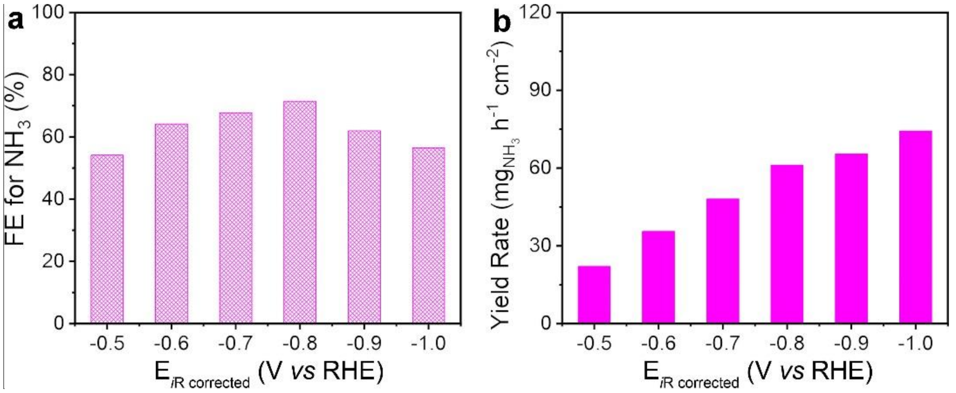

Supplementary Fig. 27. Catalytic performance of physical mixing of Cu_1-N-C and Co_3O_4/N-C. (**a**) FE for NH_3 and (**b**) yield rate of NH_3 over physical mixing of Cu_1-N-C and Co_3O_4/N-C at different applied potentials with 1 M NO_3^-.

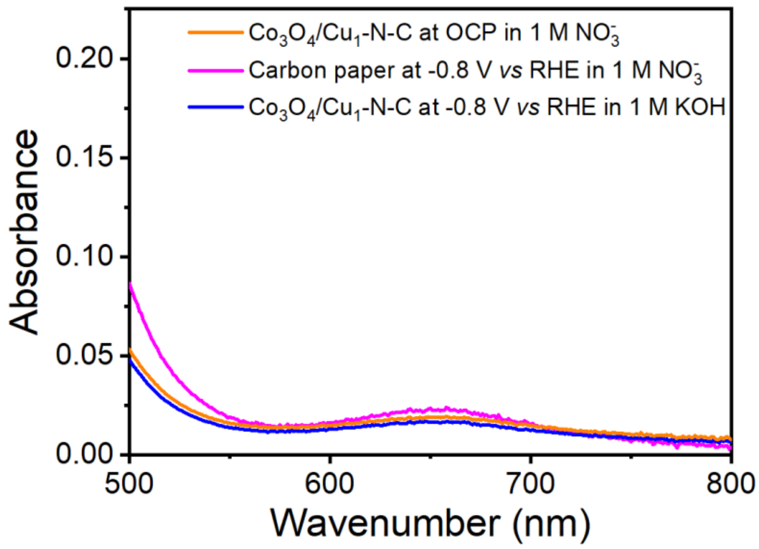

Supplementary Fig. 28. UV-vis absorption spectra of the electrolyte under different conditions.

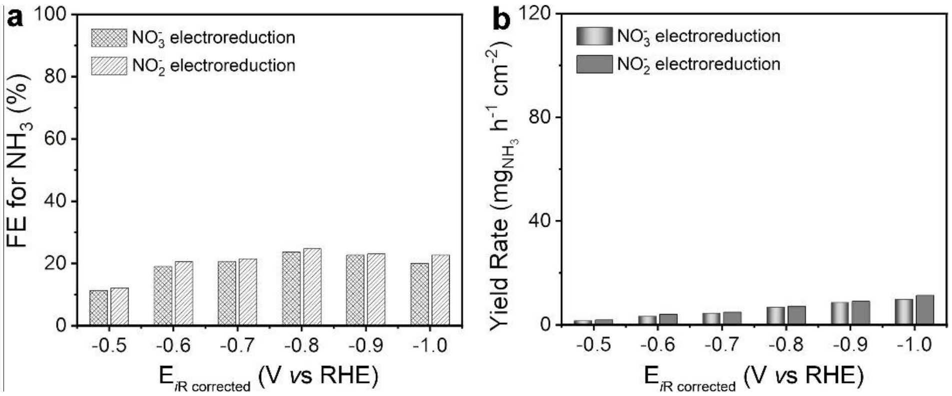

Supplementary Fig. 29. Catalytic performance of N-doped carbon. (**a**) FE for NH_3 and (**b**) yield rate for NH_3 of N-doped carbon at different applied potentials with 1 M NO_3^-/NO_2^-, respectively.

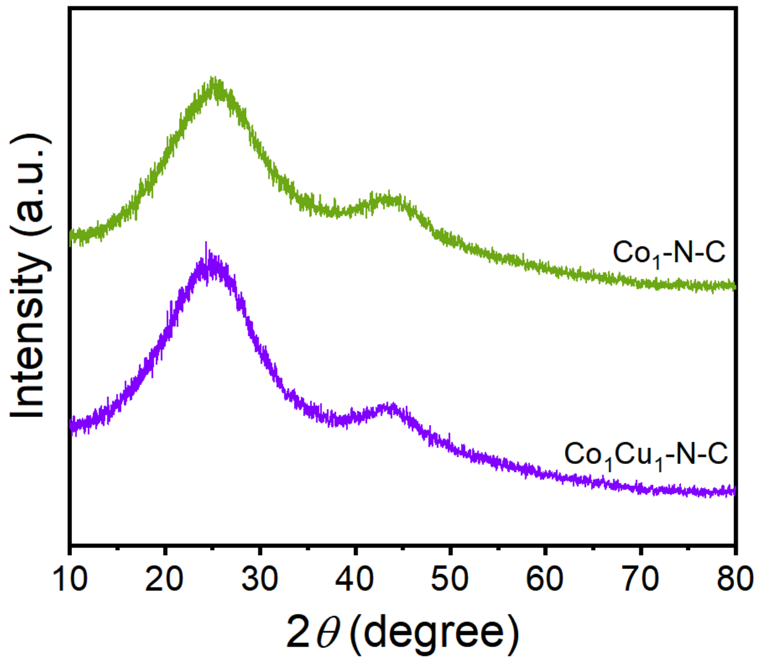

Supplementary Fig. 30. XRD patterns of Co_1-N-C and Co_1Cu_1-N-C. The XRD patterns of Co_1-N-C and Co_1Cu_1-N-C both exhibited the broad peaks which were attributed to graphite carbon. The characteristic peak of metal or metal oxides were not observed, demonstrating the absence of metal or metal oxides in the two samples.

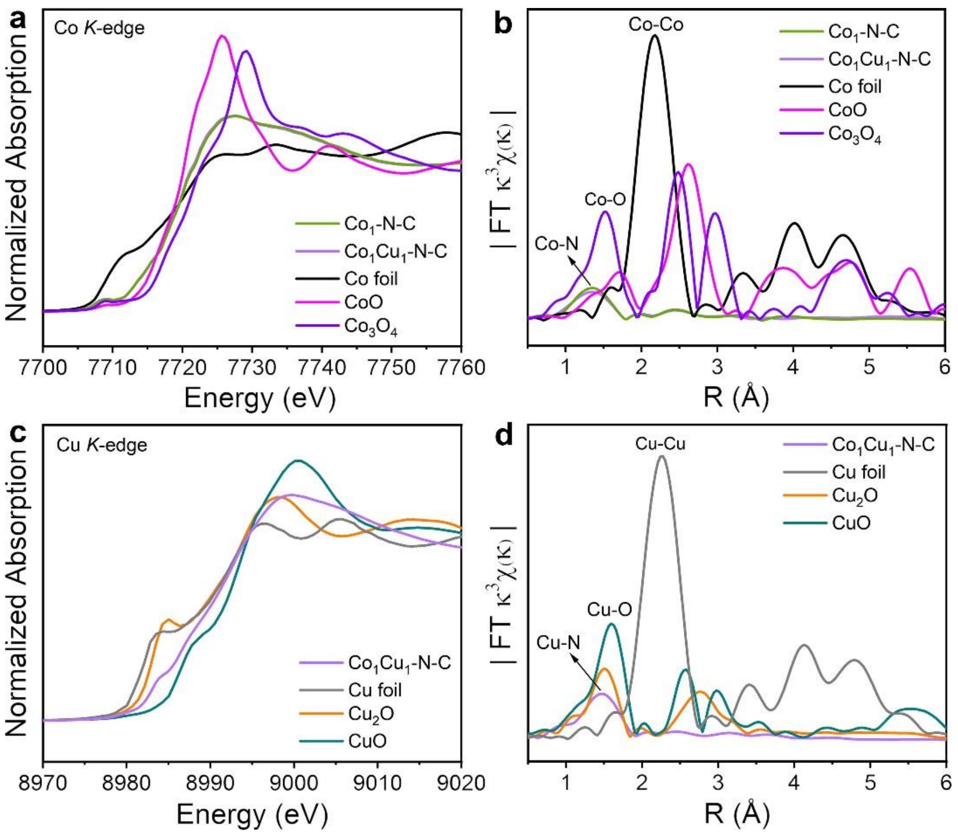

Supplementary Fig. 31. XAFS results of Co_1-N-C and Co_1Cu_1-N-C. Co *K*-edge (**a**) XANES spectra and (b) EXAFS spectra for Co_1-N-C, Co_1Cu_1-N-C, Co foil, CoO, and Co_3O_4. Cu *K*-edge (**c**) XANES spectra and (**d**) EXAFS spectra for Co_1Cu_1-N-C, Cu foil, Cu_2O, and CuO. The Co *K*-edge XANES spectra of Co_1-N-C and Co_1Cu_1-N-C both exhibited that the valence state of Co species were between +2 to +3. The absence of Co-Co bond in Co_1-N-C and Co_1Cu_1-N-C further confirmed the atomic dispersion of Co species. Besides, the absence of Cu-Cu bond in the Cu *K*-edge EXAFS spectra of Co_1Cu_1-N-C confirmed the atomic dispersion of Cu species in Co_1Cu_1-N-C. These results indicate that Co_1-N-C and Co_1Cu_1-N-C catalysts have been successfully prepared.

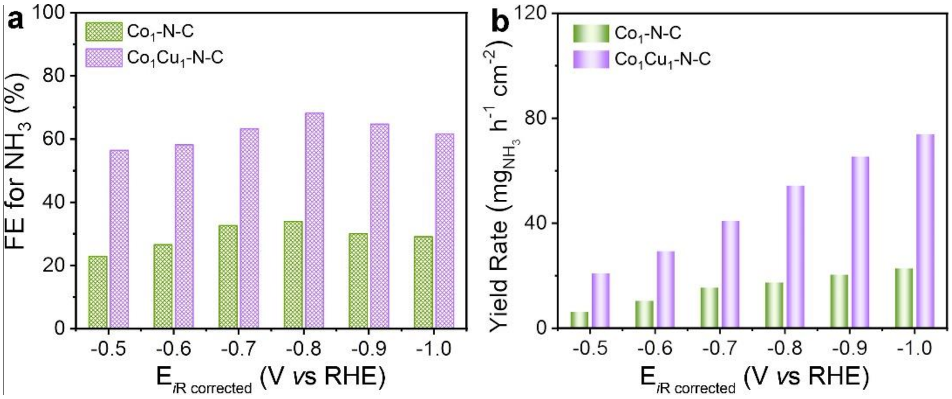

Supplementary Fig. 32. Catalytic performance of Co_1-N-C and Co_1Cu_1-N-C. (**a**) FE for NH_3 and (**b**) yield rate for NH_3 of Co_1-N-C and Co_1Cu_1-N-C at different applied potentials with 1 M NO_3^-. The interior catalytic performance over Co_1Cu_1-N-C than Co_3O_4/Cu_1-N-C suggests that synergetic role of Co single atoms and Cu single atoms were quite weak.

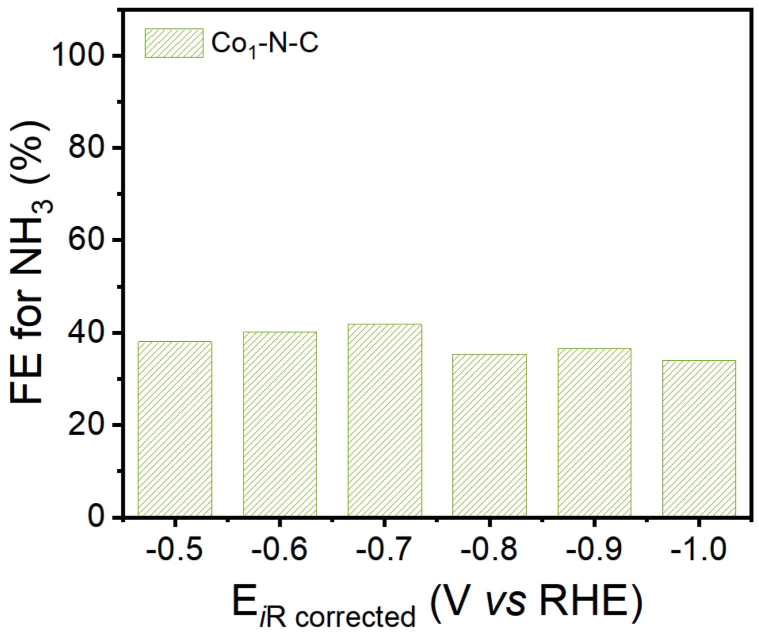

Supplementary Fig. 33. FE for NH_3 of Co_1-N-C at different applied potentials with 1 M NO_2^-. The FE for NH_3 of Co_1-N-C toward NO_2^- electroreduction was much lower than that of Co_3O_4/N-C, indicating the sluggish conversion of NO_2^- over Co single atoms.

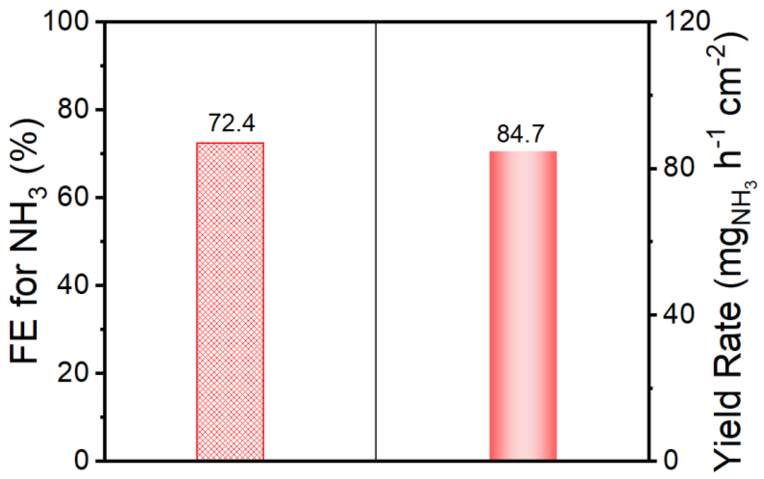

Supplementary Fig. 34. The FE and yield rate for NH_3 of Co_3O_4/Cu_1-N-C without the magnetic stirring at -1.0 V *vs* RHE.

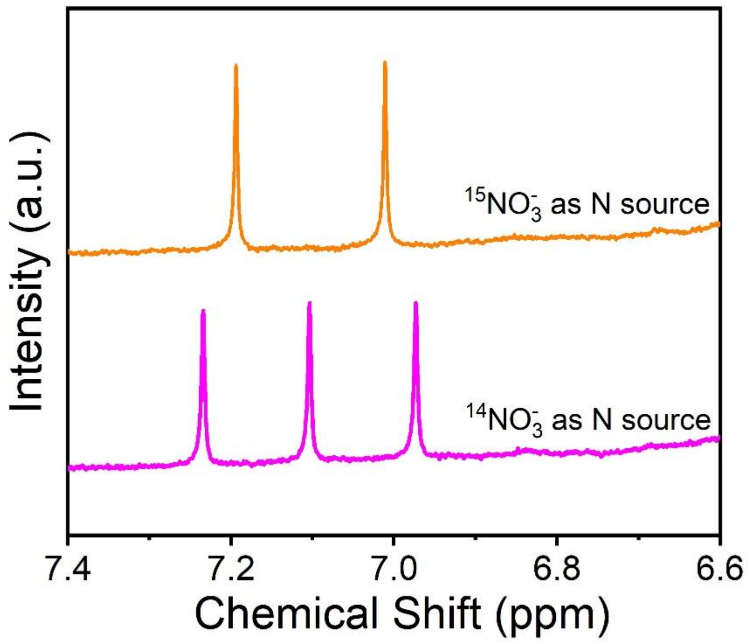

Supplementary Fig. 35. ^1H NMR spectra of the electrolyte after electroreduction reaction over Co_3O_4/Cu_1-N-C using $^{15}NO_3^-$ and $^{14}NO_3^-$ as the nitrogen source.

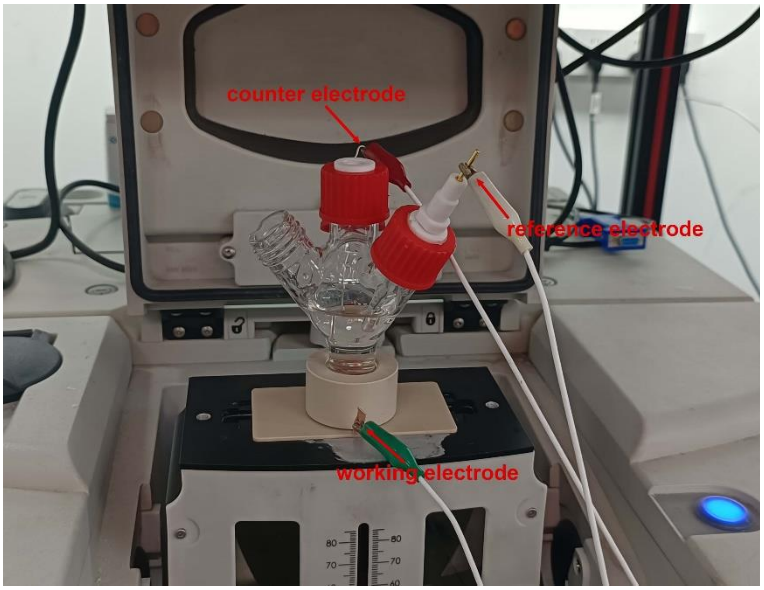

Supplementary Fig. 36. A photograph of the homemade in situ FTIR cell during the operation.

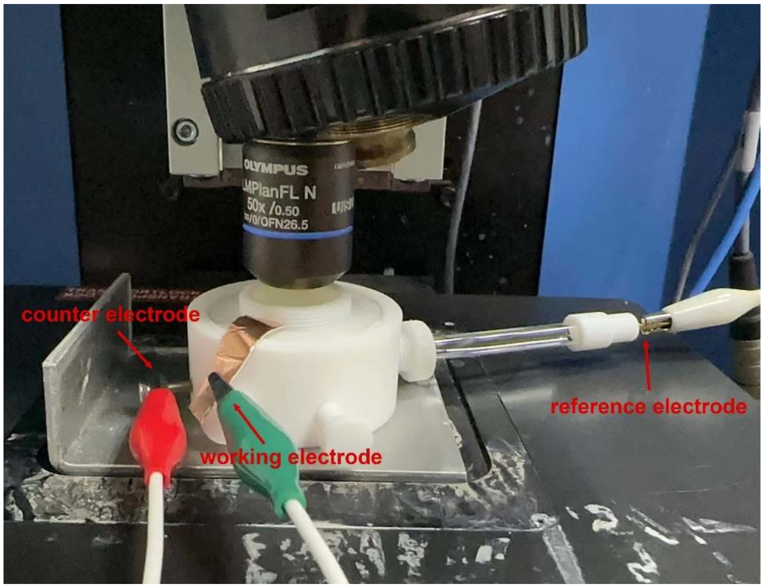

Supplementary Fig. 37. A photograph of the homemade in situ Raman cell during the operation.

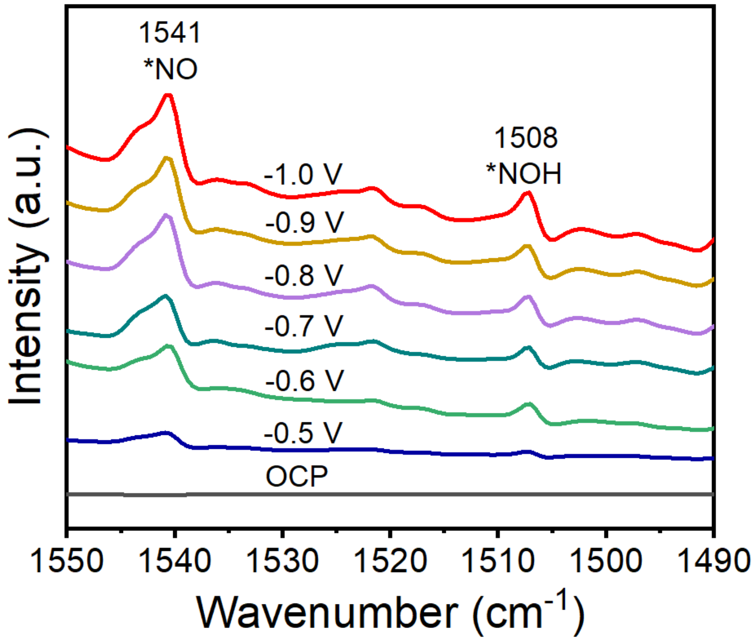

Supplementary Fig. 38. ***In situ*** **FTIR spectra for Co_3O_4/Cu_1-N-C from OCP to -1.0 V *vs* RHE in 1 M NO_3^-.**

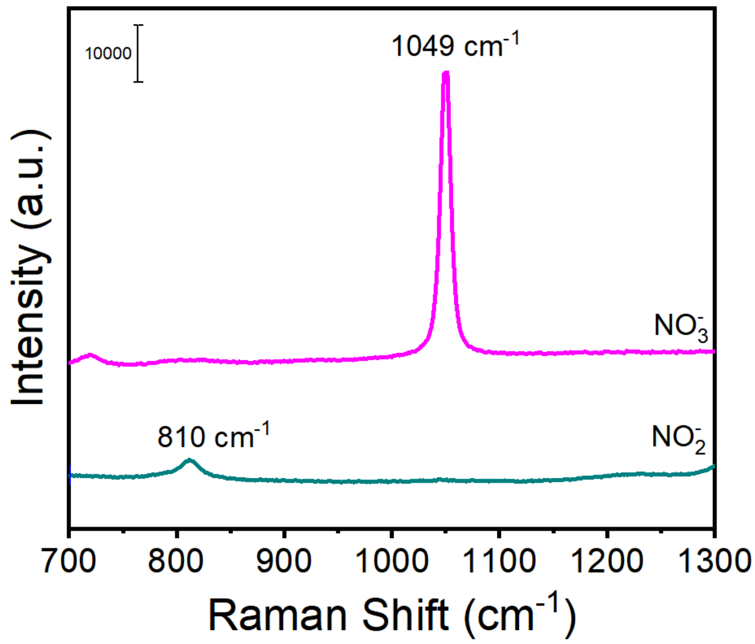

Supplementary Fig. 39. Raman spectra of 1 M KNO_3 and 1 M KNO_2 electrolyte. The ratio of the integrated area of NO_2^- to NO_3^- was determined as a correction factor (0.128) to calculate the local concentration of NO_2^-.

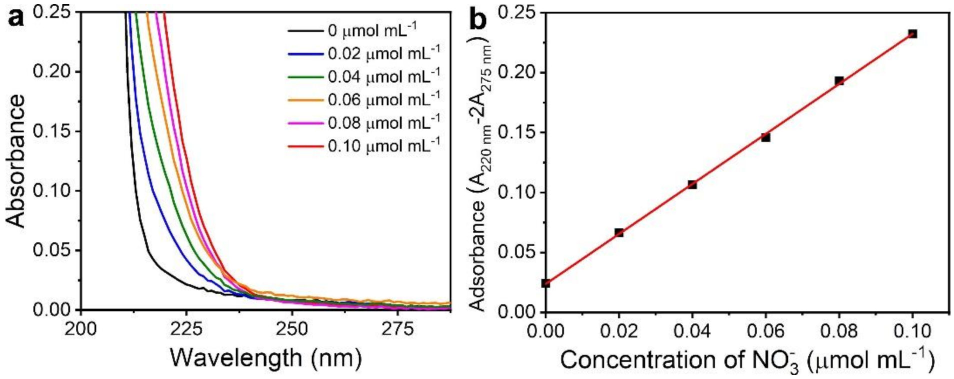

Supplementary Fig. 40. Determination of NO_3^-. (a) UV-vis curves and (b) concentration-absorbance curve of NO_3^- solution with a series of standard concentrations. The standard curve showed linear relation of absorbance with NO_3^- concentration (y = 2.084 x + 0.024, R^2 = 0.9992).

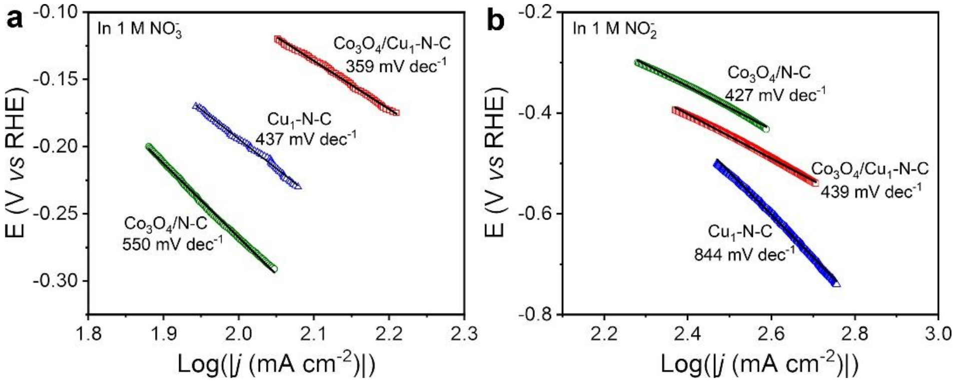

Supplementary Fig. 41. Tafel slopes of Cu_1-N-C, Co_3O_4/N-C, and Co_3O_4/Cu_1-N-C in (a) NO_3^- and (b) 1 M NO_2^-. In 1 M NO_3^-, the Tafel slopes of Co_3O_4/Cu_1-N-C was smaller than those of Co_3O_4/N-C and Cu_1-N-C. This result implies that Co_3O_4/N-C facilitated the kinetics of NO_3^- reduction. In the case of 1 M NO_2^-, the Tafel slopes of Co_3O_4/Cu_1-N-C was close to that of Co_3O_4/N-C but lower than that of Cu_1-N-C, indicating a faster NO_2^- reduction kinetics on Co_3O_4 species. As such, the combination of Co_3O_4 with Cu_1-N-C also promoted the kinetics of the subsequent NO_2^- reduction.

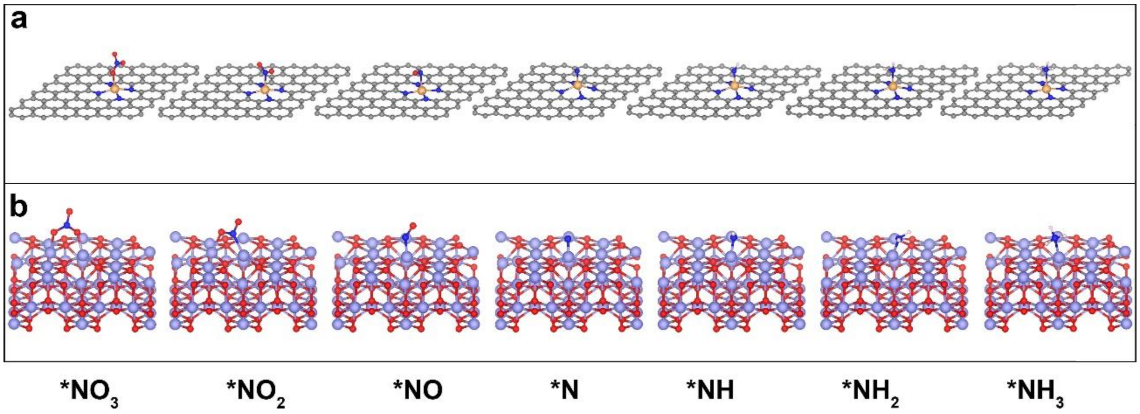

Supplementary Fig. 42. The structure models of intermediates on (a) CuN_4 and (b) Co_3O_4 (100) slabs. The gray, blue, red, white, yellow, and purple spheres represent C, N, O, H, Cu, and Co atoms, respectively.

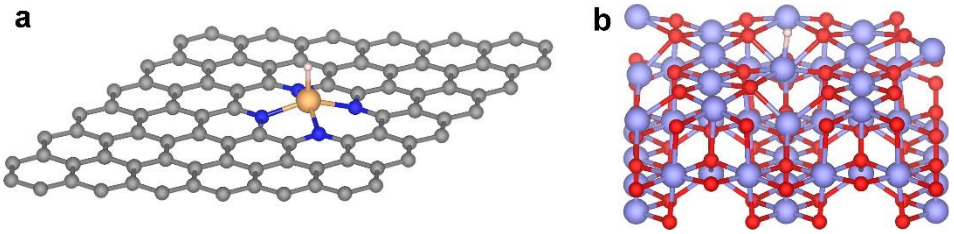

Supplementary Fig. 43. The structure models of *H adsorbed on (a) CuN_4 and (b) Co_3O_4 (100) slabs. The gray, blue, red, white, yellow, and purple spheres represent C, N, O, H, Cu, and Co atoms, respectively.

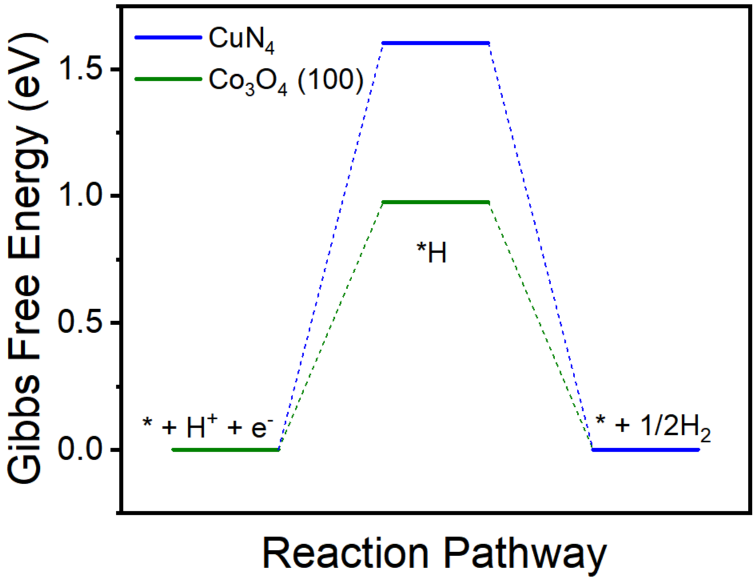

Supplementary Fig. 44. Gibbs free energy diagrams of the *H adsorption on CuN_4 and Co_3O_4 (100).

References

1 Wang, Y. *et al.* Enhanced nitrate-to-ammonia activity on copper-nickel alloys via tuning of intermediate adsorption. *J. Am. Chem. Soc.* **142**, 5702-5708, (2020).

2 He, W. *et al.* Splicing the active phases of copper/cobalt-based catalysts achieves high-rate tandem electroreduction of nitrate to ammonia. *Nat. Commun.* **13**, 1129, (2022).

3 Liu, H. *et al.* Efficient electrochemical nitrate reduction to ammonia with copper-supported rhodium cluster and single-atom catalysts. *Angew. Chem. Int. Ed.* **61**, e202202556, (2022).

4 Yang, J. *et al.* Potential-driven restructuring of cu single atoms to nanoparticles for boosting the electrochemical reduction of nitrate to ammonia. *J. Am. Chem. Soc.* **144**, 12062-12071, (2022).

5 Wang, J. *et al.* Electrocatalytic reduction of nitrate to ammonia on low-cost ultrathin coo_x nanosheets. *ACS Catal.* **11**, 15135-15140, (2021).

6 Chen, F. Y. *et al.* Efficient conversion of low-concentration nitrate sources into ammonia on a ru-dispersed cu nanowire electrocatalyst. *Nat. Nanotechnol.* **17**, 759-767, (2022).

7 Gao, P. *et al.* Schottky barrier-induced surface electric field boosts universal reduction of nox-in water to ammonia. *Angew. Chem. Int. Ed.* **60**, 20711-20716, (2021).

8 McEnaney, J. M. *et al.* Electrolyte engineering for efficient electrochemical nitrate reduction to ammonia on a titanium electrode. *ACS Sustain. Chem. Eng.* **8**, 2672-2681, (2020).

9 Harmon, N. J. *et al.* Intrinsic catalytic activity of carbon nanotubes for electrochemical nitrate reduction. *ACS Catal.* **12**, 9135-9142, (2022).

10 Wu, Z.-Y. *et al.* Electrochemical ammonia synthesis via nitrate reduction on fe single atom catalyst. *Nat. Commun.* **12**, 2870, (2021).

11 Jia, Y. *et al.* Efficient nitrate-to-ammonia electroreduction at cobalt phosphide nanoshuttles. *ACS Appl. Mater. Interfaces* **13**, 45521-45527, (2021).

12 Li, J. *et al.* Efficient ammonia electrosynthesis from nitrate on strained ruthenium nanoclusters. *J. Am. Chem. Soc.* **142**, 7036-7046, (2020).

13 Fan, K. *et al.* Active hydrogen boosts electrochemical nitrate reduction to ammonia. *Nat. Commun.* **13**, 7958, (2022).

14 Zhu, J.-Y. *et al.* Iridium nanotubes as bifunctional electrocatalysts for oxygen evolution and nitrate reduction reactions. *ACS Appl. Mat. Interfaces* **12**, 14064-14070, (2020).

15 Zhang, N. *et al.* Governing interlayer strain in bismuth nanocrystals for efficient ammonia

electrosynthesis from nitrate reduction. *ACS Nano* **16**, 4795-4804, (2022).

16 Gao, Q. *et al*. Synthesis of core/shell nanocrystals with ordered intermetallic single-atom alloy layers for nitrate electroreduction to ammonia. *Nat. Synth.* **2**, 624-623 (2023).
